# Surface engineering at the nanoscale: A way forward to improve coronary stent efficacy

**DOI:** 10.1063/5.0037298

**Published:** 2021-06-01

**Authors:** Aleena Mary Cherian, Shantikumar V. Nair, Vijayakumar Maniyal, Deepthy Menon

**Affiliations:** 1 Amrita Centre for Nanosciences and Molecular Medicine, Amrita Vishwa Vidyapeetham, Ponekkara P.O. Cochin 682041, Kerala, India; 2 Department of Cardiology, Amrita Institute of Medical Science and Research Centre, Amrita Vishwa Vidyapeetham, Ponekkara P.O. Cochin 682041, Kerala, India

## Abstract

Coronary in-stent restenosis and late stent thrombosis are the two major inadequacies of vascular stents that limit its long-term efficacy. Although restenosis has been successfully inhibited through the use of the current clinical drug-eluting stent which releases antiproliferative drugs, problems of late-stent thrombosis remain a concern due to polymer hypersensitivity and delayed re-endothelialization. Thus, the field of coronary stenting demands devices having enhanced compatibility and effectiveness to endothelial cells. Nanotechnology allows for efficient modulation of surface roughness, chemistry, feature size, and drug/biologics loading, to attain the desired biological response. Hence, surface topographical modification at the nanoscale is a plausible strategy to improve stent performance by utilizing novel design schemes that incorporate nanofeatures via the use of nanostructures, particles, or fibers, with or without the use of drugs/biologics. The main intent of this review is to deliberate on the impact of nanotechnology approaches for stent design and development and the recent advancements in this field on vascular stent performance.

## INTRODUCTION

Atherosclerosis, a chronic inflammatory condition, occurs due to the build-up of fatty deposits within coronary arteries, which causes arterial narrowing, thereby hampering blood flow.^1,2^ The advent of percutaneous coronary intervention procedures that commenced with balloon angioplasty and presently intravascular stenting has relieved millions suffering from this coronary artery disease.^3–5^ Bare-metal stents (BMS) and drug-eluting stents (DES) are the two successful intravascular stent candidates that have revolutionized the field of interventional cardiology. Although BMS could restore occluded arteries and promote re-endothelialization of the denuded artery, it suffers from in-stent restenosis in nearly 30% of the cases.^6,7^ Early restenosis occurred due to the migration and hyperproliferation of vascular smooth muscle cells (VSMCs) into the intimal space in response to vascular injury, which is caused by stent deployment.^8–11^ DES, the current clinical gold standard, evolved from BMS, which elutes an antiproliferative drug that reduced SMC hyperplasia within the stent, thereby reducing restenosis rates.^12^ DES could significantly reduce the complications of in-stent restenosis, but long-term trial revealed another challenge, which is late and very late stent thrombosis.^13–15^ The antiproliferative effect of DES slows the process of re-endothelialization, thus triggering platelet activation and late stent thrombosis.^16,17^ Even though the incidence of stent thrombosis is low, it occurs suddenly with acute life-threatening symptoms and high mortality.^15,18^ Additionally, the polymeric coatings utilized for drug incorporation induce inflammatory reactions and other instability issues, augmenting its complications.^19,20^ The development of DES progressed through various generations. First-generation DES had a stainless steel stent coated with drugs, either sirolimus or paclitaxel. Paclitaxel (PTX) inhibits microtubule disassembly and interferes with the cell cycle, leading to cell cycle arrest in G0–G1 and G2-M phases.^21^ Sirolimus binds to FKBP12 and subsequently inhibits the mTOR and PI3 pathway, arresting cell cycle in the G1 phase.^22,23^ First-generation DES used synthetic polymers such as poly(ethylene-co-vinyl acetate), poly(n-butyl methacrylate) or tri-block copolymer poly(styrene-b-isobutylene-b-styrene). Observations of late stent thrombosis and inflammatory cells surrounding the stent struts pointed to polymer hypersensitivity as the major issue.^24^ Second-generation DES utilized cobalt–chromium stents with more biocompatible polymer coatings such as phosphorylcholine and co-polymer poly(vinylidene fluoride-co-hexafluoropropylene) that reduced inflammation. These stents possess decreased strut thickness, improved flexibility, deliverability, enhanced biocompatibility, and superior re-endothelialization and are now the predominant clinical stents.^25–27^ To further reduce inflammatory response, third-generation DES utilize completely bioabsorbable polymers such as poly-lactic acid (PLA), poly(lactide-co-glycolide) (PLGA), and polycaprolactone (PCL).^28^

Researchers are now keen to advance the stent technology and devise a novel stent material/surface that can promote re-endothelialization and concurrently inhibit restenosis, without altering the hemocompatibility or stent characteristics.^29,30^ In this direction, various novel schemes have been proposed and tested at lab scale or preclinically in small/large animal models, with a few taken forward to clinical trials. Among these innovations, stent surface engineering strategies that provide novel alterations in topography, chemistry, roughness, and wettability and also offer a platform for drug/biologics loading are thrust areas in coronary stent development.^31,32^ Additionally, the clinical needs of long-term safety and bio/hemocompatibility demand high standards for the choice of the stent material, design, or surface.^33,34^ Stent surface engineering is the process of modifying the surface of a stent material to enhance its overall performance characteristics. Engineering a surface for a desired outcome can be done either by chemically modifying the existing surface or by depositing a thin film with the desired properties onto the existing surface.^35–37^ This would obviate corrosion and ion leaching, improve biocompatibility and durability, and also enhance cell–material interactions. Nanosurface engineering encompasses engineering materials and/or technologies at the nanoscale, wherein one or more features are less than 100 nm in at least one dimension. This may refer to the size of individual crystals, grains, pores, particles, fiber diameter, etc.^37^ Specifically, by exploiting the high surface area to volume ratio, surface energy, roughness, reactivity, and wettability offered by nanostructures, nanosurface engineering of stents can be a way forward to design stents with improved biological performance.^38,39^ It is also necessary that the mechanical characteristics (stent deliverability, crimping and expansion profile, and coating durability) of the stents are retained without significant variations, after nanotexturing. Progress in nanotechnology now makes it possible to precisely design and modulate the surface properties of materials at the nanoscale via alterations in the method of processing, choice of the stent material, incorporation of drugs/biologics, etc.^40–42^ From the materials engineering perspective, physico-chemical properties of a biomaterial surface (namely, topographical features and roughness, surface chemistry, and hydrophilicity) can profoundly affect cellular mechanisms. Likewise, shape and size of the structures can regulate cellular functions by modifying the cytoskeleton organization. Nanoscale topographies can also critically control the signaling pathways at the molecular and subcellular levels.^43,44^ Thus, nanosurfaces can uniquely direct the overall biological response and hemocompatibility of the implanted stent material, mainly, its protein adsorption, vascular cell [smooth muscle cell and endothelial (EC) cell] adhesion and proliferation, etc.

**FIG. 1.**
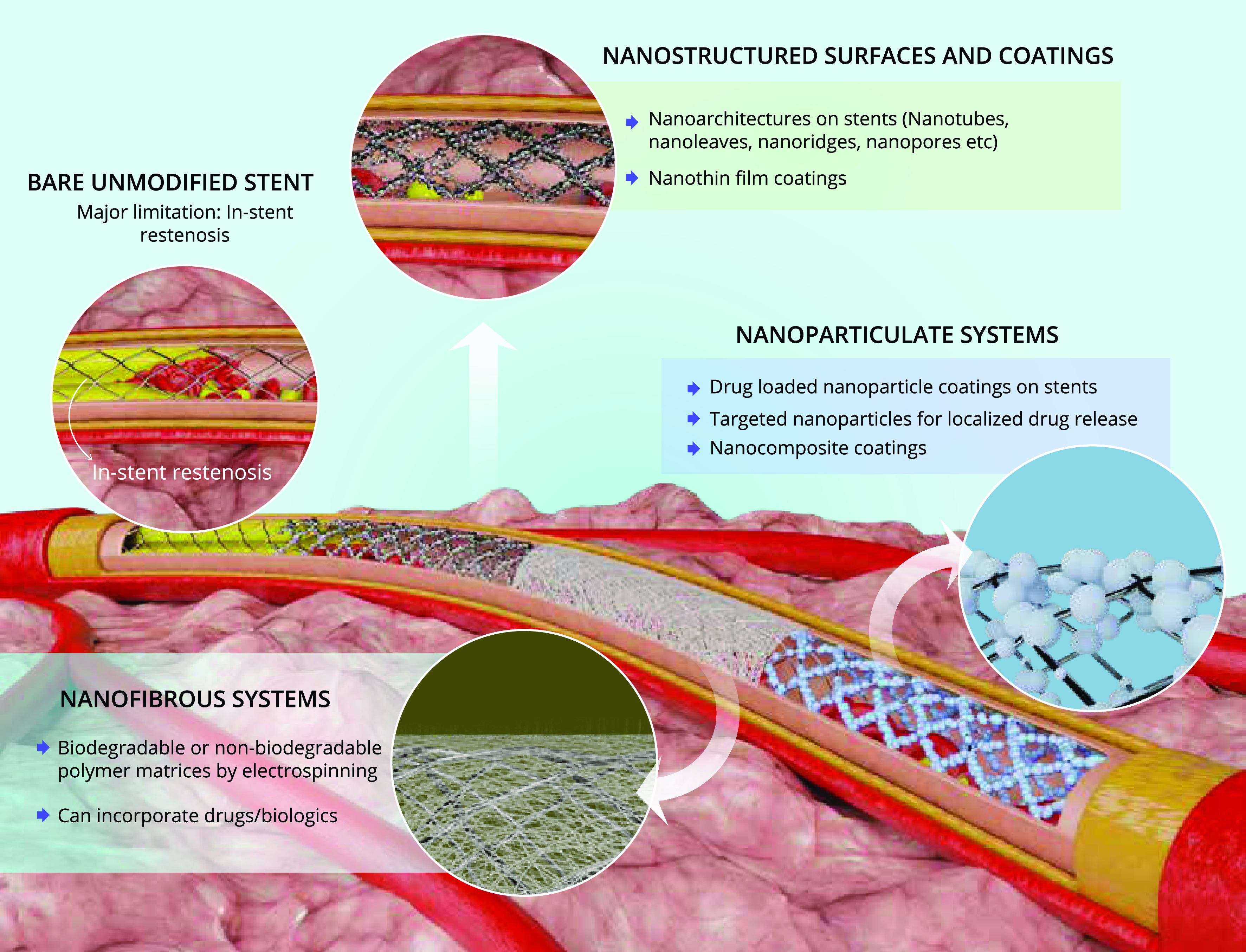
Various nanoscale surface engineering strategies (nanostructured surface and thin films, nanoparticulate, and nanofibrous) adopted as coatings on coronary bare-metal stents to prevent in-stent restenosis and promote re-endothelialization.

Recent advances encompass a paradigm shift toward nanoengineered stent coatings to improve stent efficacy, which include polymer-less techniques of stent modification, coatings for controlled drug delivery, drug-free nanotopographical approaches, and nanoparticle (NP)-eluting/nanofiber-coated stents. Nanofiber coatings on stents developed by electrospinning are classified as nanotechnology, as the electrospun fiber diameters are at the nanoscale,^45^ although it does not represent “surface engineering.” A wealth of literature exists that delves on various nanotechnology-based techniques that help to generate nanoscale surface features and coatings on existing stent materials. Such nanoengineered stents alleviate the problems of restenosis, lack of re-endothelialization, local inflammatory response, and thrombus formation,^41,46^ which are common to BMS or DES. The future generation of cardiovascular stents will be nanotechnology-centric, due to the multifarious benefits it promises. Despite these advantages, a relevant clinical translation of stents utilizing this technology in the biomedical device industry is still awaited.^47,48^ This review throws light into the diverse nanosurface modification strategies (Fig. 1) that are widely adopted for developing novel stents and their associated cellular response *in vitro* and *in vivo*. To translate the research findings from bench to bedside entails the development of a viable stent prototype and its further preclinical evaluation in animal models, followed by regulatory approval and clinical testing. The journey of those few stents that surpassed these standards to the clinical trial stage is also presented.

### Nanostructured surfaces and nano-thin-film coatings

#### Nanoscale architectures on stent

Texturing the stent surface at nanoscale may be beneficial, given the fact that nanosurface topography mimics the natural extracellular matrix and can regulate vascular cell adherence and proliferation.^49^ Cells when in contact with nanostructured surfaces not only respond to the type of material, but also to the surface topology.^50^ Surface properties such as topography and chemistry, roughness and wettability, are known to influence protein and cell adhesion.^41,51^ Moreover, creation of reservoirs and pores at nanoscale provides a platform to load drugs efficiently.^52^ Development of polymer-free stents can eliminate the problems such as polymer delamination and the long-term risk of inflammatory response, and thereby help to better endothelial regeneration.^53^ This section elaborates the diverse nanostructured surfaces on vascular stents and their biological response. Among the medically relevant metals such as titanium (Ti), magnesium (Mg), iron (Fe), and the alloys, viz., stainless steel (SS), cobalt–chromium (CC), nitinol (NiTi), etc., metallic stents are mainly based on the alloys of SS, CC, NiTi, Mg, and Fe.^54^ Significant efforts are under way to obtain nanostructured surfaces on these alloyed metals, which include the widely studied electrochemical anodization process, physical/chemical vapor deposition (CVD), thermochemical processing, lithography, etc.^55^

##### Nanotubular structures

Titanium dioxide nanotubes (titania nanotubes or TNT) can be fabricated using diverse methods including sol-gel,^56,57^ hydrothermal processes,^58–60^ template-assisted synthesis,^61,62^ seeded growth,^63^ and electrochemical anodization.^64–67^ Among all these methods, electrochemical anodization is widely used, because it provides a relatively simple and effective way of generating nanotubular structures. Moreover, it is a cost-effective process which offers the feasibility to tune the size and shape of nanotubular arrays to the desired dimensions. Furthermore, the tubes prepared via this method are highly ordered, well-defined with high aspect ratios, and are vertically oriented to the substrate, with good adherent strength.^68–70^ Most importantly, the dimensions of nanotubes such as diameter, length, wall thickness, etc., can be controlled precisely and modified easily by modulating the anodization parameters.^71,72^ However, such nanotubes have mostly been developed on metallic Ti or NiTi surfaces by anodization in acidic or alkaline conditions at different applied voltages for varied time intervals.^73,74^ By changing the anodization parameters (applied voltage and anodization time), TiO_2_ nanotubes having different diameters from 15 to 300 nm, and different lengths (nm to mm) can be obtained.^75^ The excellent potential of titanium nanotubes is mainly due to its high effective surface and the possibility to vary their geometry (diameter and length), which could be specifically designed for a desired biological response.^74^

Titanium, due to its low tensile strength and ductility, failed to make an impact as a sole stent material, because of its higher probability of tensile failure upon expansion when developed as stents.^76,77^ Despite these limitations, immense information has been garnered on the impact of nanoscale dimensions in modulating vascular cell behavior *in vitro* by utilizing anodized Ti surfaces. An optimal surface engineered stent should inhibit vascular smooth muscle cell proliferation to prevent in-stent restenosis, but simultaneously enhance endothelial cell adhesion and proliferation for restoration of a healthy endothelium.^78^ Several studies report TNTs as a promising platform for cardiovascular stent applications owing to their selective regulation of vascular cell response, specifically endothelial (EC) and smooth muscle cells (SMC), but the reason for this selective cell proliferation is still unknown and not clearly elucidated by researchers. The cell viability and activity on a nanostructured surface depends on various parameters such as the surface topography and roughness, surface wettability, and surface chemistry, which collectively dictate the cell response.^39^ Recently, a study was conducted to investigate the combined influence of nanotopography and surface chemistry on the *in vitro* biological response of TiO_2_ nanotubes. It was observed that nanotopography, surface chemistry, and wettability as well as morphology, cooperatively contributed to the reduced platelet adhesion and preferential vascular cell response.^79^ Several other studies report that TiO_2_ nanotubes of varied nanotube diameters (30–90 nm) promoted EC growth and proliferation, with concurrent inhibition of smooth muscle cells.^80,81^ The highlights of such *in vitro* studies include faster migration of ECs on nanotubular surface^82^ and lower inflammatory response, resulting in reduced TNFα-induced SMC proliferation,^83^ good hemo- and cytocompatibility with lessened platelet adhesion, and enhanced endothelial cell adhesion and proliferation for smaller diameter (30 nm) nanotubes.^84^ Rapid re-endothelialization, a key to the success of a cardiovascular implant device, has been achieved through several synergistic approaches on TiO_2_ nanotubular surfaces. Fibronectin (Fn), an extracellular matrix protein, when immobilized onto TNTs via an intermediate polydopamine (PDA) layer, has offered increased nitric oxide and prostaglandin (PGI_2_) secretion, indicating an increased functionality of ECs on these surfaces.^85^ Likewise, TNTs functionalized with polydopamine (PDA/NTs) showed a remarkable enhancement in the mobility of ECs with longer migration distances than that of bare Ti and TNTs, respectively.^86^ PDA/NTs incorporating a thrombin inhibitor, bivalirudin (BVLD), demonstrated high BVLD elution beyond 70 days. This synergism brought about a significant inhibitory effect on thrombin bioactivity, with concomitant less adhesion, activation, and aggregation of platelets, and selectivity for EC over SMC in a competitive growth environment.^87^ Recently, utilizing copper as a catalyst for effecting the release of nitric oxide from endogenous nitric oxide donors, Cu-loaded PDA nanoparticles were stacked onto TNTs. This surface yielded a controlled and steady release of Cu, sufficient to enable the release of NO within the physiological range. The *in vivo* effect induced by this synergy aided in preventing intimal hyperplasia and coagulation, with simultaneous rapid re-endothelialization after implantation in the abdominal aorta of rats.^88^

Another study utilized a nanotubular oxide layer as a drug reservoir on anodized Ti-8Mn alloy as a nickel and polymer-free matrix for drug-eluting stents. The highly ordered Ti-8Mn oxide NTs promoted cell (neonatal mice skin cells) proliferation in comparison with flat substrates. It was noted that alloying titanium with 8% manganese hindered charge transfer from fibrinogen to the material, thus preventing blood clots and thrombus formation. They demonstrated that the drug loading efficiency was higher on this alloyed nanotube surface, thus establishing Ti-8Mn oxide NTs to be a superior platform for drug loading than TNTs.^89^ Self-grown nanotubes of two nanotube morphologies, viz., homo, and hetero-NT, which are highly ordered and vertically aligned, with variations in tube diameter (80–190 nm), were developed on Ti–17Nb–6Ta substrate. Both NT morphologies showed significantly better results for endothelial cell proliferation, with homo-NTs displaying superior biological activity and drug loading capacity than hetero-NTs.^90^

**FIG. 2.**
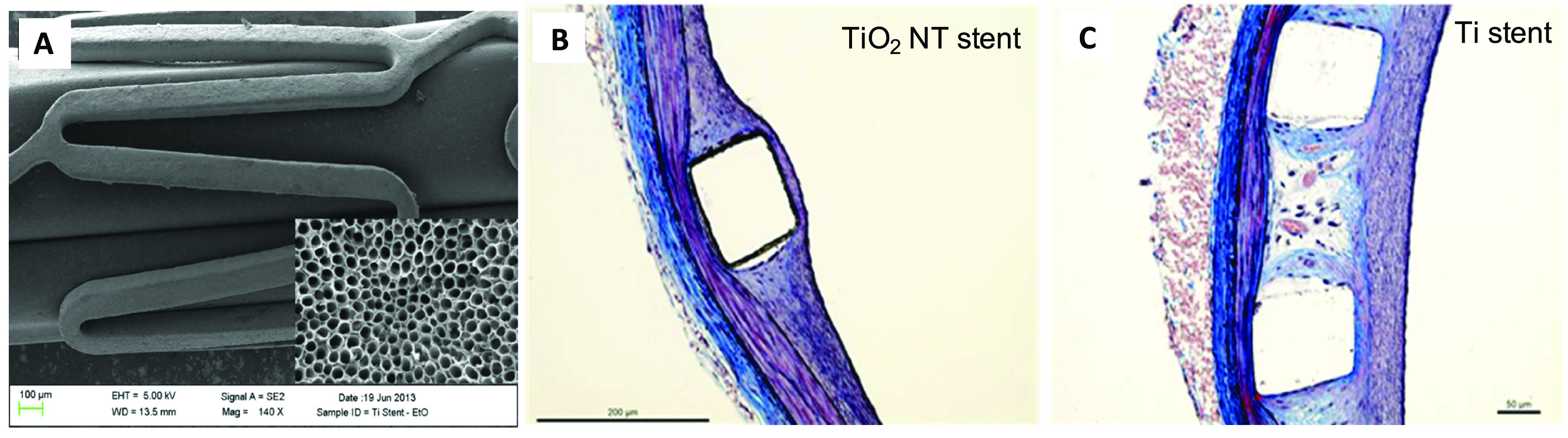
(a) Electron micrograph of titania nanotube coated stent. Inset: nanotubes with an average nanotube diameter of 90 nm (magnification ×250 000). Moffat trichrome-stained images of a stented artery. (b) Titania nanoengineered and (c) Ti stents, showing a 15.6% and 5.6% thinner neointima over the struts for TiO_2_ NT stents than Ti stent. Reprinted with permission from Nuhn *et al.*, ACS Appl. Mater. Interfaces **9**(23), 19677–19686 (2017). Copyright 2017 American Chemical Society.

Despite the abundant literature on TNT-based systems for cardiovascular stenting, no studies have yet proven the utility of this material for clinical translation. This could be due to the limitations of Ti as the base material for stent manufacturing and the complexity involved in translating TiO_2_ nanostructures onto clinically available coronary stent materials like SS and CC. This requires that titanium, which is deposited on SS or CC stents, be anodized to generate TNTs. Here, the restraints posed by the process of anodization (strong acidic/alkaline environment) can hamper the durability of the extremely thin stent struts (typically <100 *μ*m) upon expansion and crimping. In a sole *in vivo* study reported thus far on a titanium stent prototype (Ti6Al4V) bearing nanotopographical cues of diameter 90 ± 5 nm and height 1800 ± 300 nm, significantly lower restenosis rates with minimal intimal hyperplasia and good stent strut coverage were observed after implantation in rabbit iliofemoral arteries as depicted in Fig. 2. This ascertained the importance of nanotopography in offering reduced in-stent restenosis, with concurrently enhanced endothelialization.^91^

In contrast to titanium-based stents, which find minimal use in coronary stenting, nitinol (NiTi), a widely explored alloy of titanium with nickel, finds applicability as coronary stents. The process of anodization helps to generate Ni–Ti–O nanotubular structures on the NiTi surface. The impact of nanotopography on vascular response to nitinol substrates was akin to titanium nanotubular structures when investigated *in vitro* using ECs and SMCs. These NTs showed reduced proliferation of SMCs, along with a decreased expression of collagen I and MMP-2, and parallelly enhanced EC spreading and migration.^92^ Moreover, nanotube diameter was found to influence EC and SMC response. While SMCs proliferated less, ECs showed increased proliferation and migration, with augmented production of elastin and collagen, on larger diameter (110 nm) NTs.^93^ Regardless of the limited investigations done on NiTi surfaces, nanotopography, especially NTs, showed promise for stenting applications and surprisingly these nanostructures always exhibited a preferential vascular response, the reason for which remains to be explored. Researchers have also developed a nanotubular α-Fe_2_O_3_ coating on biodegradable iron stents. PLGA coated on the NT surface incorporating the drug (Rapamycin) could efficiently reduce the initial burst with a sustained drug release of 30 days. These surfaces showed better EC viability than SMC along with good hemocompatibility.^94^

##### Other nanotopographies (nanoleaves, nanograss, nanoflakes, nanopillars, and nanowires) on stent surface

**FIG. 3.**
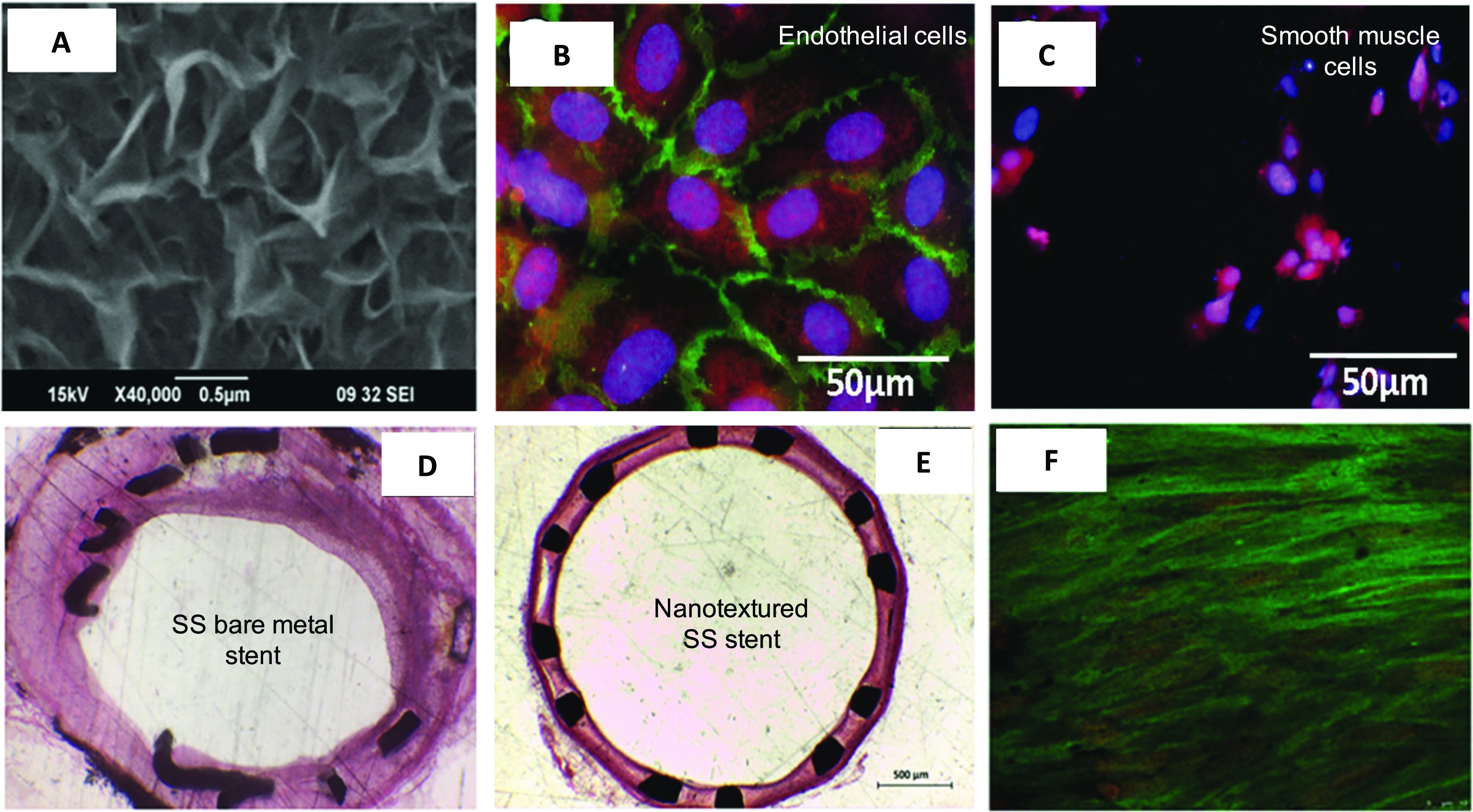
(a) Electron micrograph of titania nanoleafy textured stent surface (magnification ×40 000). Fluorescence imaging of (b) endothelial cells stained for F-actin (red) and PECAM1 (green) and (c) smooth muscle cells stained for F-actin (red) and nucleus (blue) on nanotextured SS surfaces, showing preferential adsorption and proliferation of ECs over SMCs on nanoleafy SS surface. Reprinted with permission from Mohan *et al.*, Adv. Healthcare Mater. **6**, 1601353 (2017). Copyright 2017 John Wiley and Sons. H&E images of rabbit iliac artery after 2 months implantation of (d) SS bare-metal stent and (e) nanotextured SS stent, showing nearly 50% decrease in neointimal stenosis for the nanotextured stent. (f) Immunofluorescent en-face stained images of wheat germ agglutinin on ECs in nanotextured stent implanted artery, showing complete endothelialization (scale bar: 10 *μ*m). (a) and (d)–(f) Reprinted with permission from Cherian *et al.*, ACS Omega **5**, 17582–17591 (2020). Copyright 2020 American Chemical Society.

As an alternative to anodization, researchers have delved into chemical/thermochemical processing or lithography^95,96^ as a means to develop uniform and homogeneous nanostructures on metallic surfaces.^31^ Hydrothermal/thermochemical synthesis proposes various advantages such as low cost, simple experimental set up, and high yield.^97^ In a normal hydrothermal reaction, acidic or alkaline media are subjected to elevated temperature and pressure for a specific time, thus providing a one-step process for the generation of highly crystalline materials.^98^ The reaction parameters such as concentration and type of solvent, reaction temperature and time, offer significant effects on the formed nanostructures.^99,100^ Diverse titania nanotopographies were generated on Ti substrates using this facile thermochemical technique in NaOH at 200 °C, which exhibited a preferential vascular cell response, like for titania NTs.^101^ Static and dynamic blood contact studies done on Ti stent prototypes revealed these hydrothermally generated nanostructures to be antihemolytic, with minimal activation of coagulation cascade and platelets.^102^ Among the different topographies, a specific titania nanoleafy structure [Fig. 3(a)] yielded superior cyto- and hemocompatibility response *in vitro*, with high endothelialization and low SMC proliferation [Figs. 3(b) and 3(c)]. The uniqueness of this simple polymer-free and drug-free nanotexturing approach is that it could be readily translated onto any metallic stent substrate or on clinical stent materials of SS and CC. The nanoleafy structures were found to be extremely stable and adherent upon stent crimping and expansion with good corrosion resistance.^103^ This titania nanotexturing developed on SS bare-metal coronary stents presented minimal in-stent restenosis, effective endothelialization, and no thrombus formation after 8 weeks of implantation in a rabbit iliac artery model,^104^ as evident from Figs. 3(d)–3(f).

Likewise, direct nanotexturing of metallic substrates have also yielded nanotopographical features. For example, nanosized pyramidal structures were developed on SS substrates by hydrothermal treatment under alkaline conditions, which showed improved corrosion resistance, hemocompatibility, and EC growth, while inhibiting the proliferation of SMCs.^105^ A superhydrophilic nanoscale morphology with nanograss-like structures was likewise generated on Ni-free Ti–29Nb alloy after subjecting it to hydrothermal processing in alkaline sodium hydroxide solution at 250 °C for 10 h. This nanostructured material showed reduced hemolysis, minimal platelet adhesion, and activation upon contact with blood. The initially adsorbed intermediate water layer on this superhydrophilic surface might have caused resistance to platelet attachment, which can be attributed to the existence of a large number of hydrogen bonds.^106^ In the same manner, radially emanating metallic nanopillar structures were created on the surface of CC stent wires (MP35N) via controlled RF plasma processing technique.^107^ These uniformly coated nanopillar arrays of diameter 100 to 300 nm were developed directly on the stent wires. This surface displayed greater endothelial cell growth and functionality, continuous and complete endothelial monolayer formation, and minimal oxidative stress level in ECs.^108^ Increased EC and SMC adhesion has also been reported on nanostructured Ti and CC surfaces generated by compacting commercially pure metal particulates. Well-spread morphologies of both vascular cell types, with an increased ratio of viable ECs to SMCs, were noted on these nanostructured surfaces. A large number of particle boundaries at the surface of nanostructured metals were speculated to be responsible for improved adhesion of vascular cells on these surfaces.^109^ Such nanostructured Ti also showed greater competitive adhesion of ECs than SMCs.^110,111^ Utilizing a simple chemical conversion treatment of Mg−Nd−Zn−Zr alloys in 0.1 M potassium fluoride solution, they were surface textured to deposit MgF_2_ film with nanoscale flake-like features (∼200–300 nm-sized, having a thickness of 800 nm). These nanotextured films showed a significant reduction in corrosion rates and presented a favorable surface for enhanced viability, growth, and proliferation of ECs. Furthermore, implantation in rabbit abdominal aorta confirmed a complete and uninterrupted endothelial lining on the nano-MgF_2_ modified stent, along with minimal inflammatory reaction, thrombogenicity, and restenosis.^112^ Similar to the above studies, ultra-thin (300 nm) and chaotic one-dimensional (1D) aluminum oxide (Al_2_O_3_) nanostructures having a nanowire (NW) morphology were synthesized by chemical vapor deposition on a glass substrate. Al_2_O_3_ NWs presented a preference for EC adhesion and proliferation in comparison with SMC.^113^ ECs seem to favor growth on low-density NWs, while SMCs disliked this topography in contrast to commercially available microstructured Al_2_O_3_ plates.^114^ Recombinant filamentous bacteriophages (re-phage) with a cell adhesive peptide (RGD) were immobilized onto Al_2_O_3_ NWs by a simple dip-coating process for improvement of cell binding. This re-phage-coated material allowed a strong EC–nanostructure interaction, with increased cell population and viability, in comparison with Al_2_O_3_ NWs.^115^ A novel superhydrophobic hybrid coating that couples the effect of the topography of Al_2_O_3_ NWs and the low surface energy of poly (bis (2,2,2-trifluoroethoxy) phosphazene) (PTFEP) was developed by chemical vapor deposition (CVD) method and ultrasonic infiltration technique, for improved hemocompatibility of cardiovascular implants. The dual-scale surface roughness (micro/nano) and the superhydrophobic nature of the nanowired substrate reduced the contact area between the surface and blood, yielding a non-wetting surface that prevented platelet adhesion and activation. This reduced contact area and non-wetting nature imparted significant blood repellence to the surface.^116^

The impact of surface roughness in modulating EC response has also been investigated by various groups, especially on Ti surfaces. It is generally noted that ECs interact more efficiently on nanometer rough surfaces than on flat surfaces, with enhanced adhesion, proliferation, and migration. Nanoscale surface roughness on Ti enabled better and well-adherent endothelium under flow conditions as well.^117^ Such nanotexturing approaches have also been translated to metallic alloys and polymers. Commercially pure titanium, titanium alloy (Ti6Al4V), and polymers used to incorporate drugs in DES (e.g., polyethylene terephthalate, polytetrafluoroethylene, polyvinyl chloride, polyurethane, and nylon) were modified using an ionic plasma deposition and nitrogen ion implantation plasma deposition process to generate nanorough surface features. It was demonstrated that changes in surface chemistry and roughness at the nanoscale resulted in improved adhesion of ECs.^118^

Thus, results from literature point to the fact that nanostructured surfaces, irrespective of the method used or its surface chemistry, are able to modulate vascular cell response preferentially, with specific nanotopographies favoring endothelial cell adhesion and proliferation over smooth muscle cells. Various studies cited in this review,^103–105^ especially the titania nanotubes,^80–83^ have shown a selective response to the vascular cells, specifically ECs and SMCs. The exact reason for this preferential response still remains unclear and requires further investigation.

##### Patterned nanostructures

Femtosecond laser irradiation can produce periodic nanostructures on metals and semiconductors.^119–121^ Hierarchical micro/nanostructures possessing properties of dual-scale roughness were fabricated on Ni–Ti using a femtosecond laser for the surface modification of stents. Hydrophilic periodic nano- and hydrophobic micro/nanostructures formed could regulate the spreading of ECs, with more effect observed at the nanometer scale. Moreover, platelets failed to adhere to the micro/nanostructures.^122^ Utilizing femtosecond laser, micro-/nanobiomimetic surface patterns mimicking the morphology of VSMCs were generated on 316L SS stents. *In vitro* studies showed that this VSMC-biomimetic surface pattern of width ∼700 nm promoted adhesion, proliferation, and migration of ECs, with rapid re-endothelialization *in vivo* after 30 days.^123^ Ti patterning formed by plasma-based dry etching technique with a width and spacing varying from 750 nm to several micrometers presented significantly improved function and orientation, higher density, viability, and proliferation of rat aortic ECs compared to substrates with micro and random nanofeatures.^111^ Patterned TiO_2_ nanogratings as small as 70 nm significantly inhibited the proliferation of SMCs and concurrently enhanced EC proliferation. ECs could sense these nanogratings, yielding elongated morphologies with a larger number of focal adhesions on the patterned surfaces.^124^ Large-scale nanopatterns were developed on NiTi stents using target-ion-induced plasma sputtering (TIPS) onto which the PTFE layer was coated, resulting in a nanoporous surface having diameters ranging from 100 to 200 nm and depths of 600 nm, with infiltration of PTFE into nanoscale pores.^125^ Tantalum coating was provided on the same nanoroughened NiTi stents to generate distinct and uniform nanoscale surface structures (50–100 nm), as a means to reduce Ni ion release from the base material. These Ta-coated NiTi stents significantly improved EC attachment, proliferation, density, and coverage, in comparison with bare stents that had a considerable decrease in EC proliferation due to rapid dissolution of Ni ions.^126^ A similar process was used to develop a Ta-implanted nanoridge surface with a ripple-like surface pattern having round hills and steep valleys (depth of ∼470 nm) on CC stents. ECs that adhered on this stent surface formed numerous inter-endothelial adherent junctions through cell membrane protrusions, with faster migration rates and proliferation, besides exhibiting minimal platelet activation and fibrin formation. Synergistic effects of Ta and nanoscale surface features of this stent in rabbit iliac artery model resulted in very minimal intimal hyperplasia and lumen loss, with rapid re-endothelialization.^127^ Similarly, zirconium (Zr) ion implantation using metal vapor vacuum arc plasma source with pure Zr as the target material resulted in a nanopatterned Zr–NiTi alloy. Further, a thick Ni-depleted composite ZrO_2_/TiO_2_ nanofilm was developed on the surface of zirconium–NiTi (Zr–NiTi). Corrosion resistance was increased, depletion of Ni in the superficial surface layer resulted in reduced ion release rate of Zr–NiTi, and EC proliferation was favored after five and seven days of culture.^128^

##### Nanoporous architecture on stents for drug/biologics loading

In addition to the impact of nanotexturing on cellular response *in vitro* and *in vivo*, researchers have investigated the combined effects of nanotexturing with biologics/drug incorporation. Mostly, the nanotextures present on metallic surfaces are porous and these can be efficient sites for high drug loading.^41^ This polymer-free approach can be a viable strategy to circumvent the risks of using polymers as drug-eluting stent coatings.^129,130^ In one such study, an anti-CD146 antibody anchored onto a porous architecture bearing nanosized silicone filaments on CC stent surface helped to develop an endothelial progenitor cell (EPC) capturing stent. This stent enabled enhanced selective capture and adherence of circulating EPCs from blood and thereby induced rapid healing of endothelium at 1-week implantation in porcine, resulting in reduced neointimal thickening. Thus, the co-existence of the silicone nanofilaments and CD146 antibody provided synergistic effects for suppression of in-stent restenosis by promoting re-endothelialization.^131^ A nanoporous Al_2_O_3_ nanocoating (∼200 nm thick) on NiTi alloy substrate deposited via simple sputtering, followed by functionalization with VEGF, helped to significantly enhance EC adhesion, spreading, and proliferation. Additionally, higher levels of NO and prostaglandin (PGI_2_) secretion on the nanocoating indicated its advantage on EC functionality.^132^ Similarly, a ceramic stent coating of nanoporous alumina on SS stents served as a suitable carrier for the drug tacrolimus. These drug-coated nanoporous stents showed inhibition of neointimal proliferation in rabbits.^133^ However, after implantation in a porcine model, particle debris resulting from the cracking of ceramic coating during stent expansion resulted in increased neointimal growth and stenosis in these stents.^134^ A polymer-free sirolimus-eluting stent (PFSES) with a unique nanoporous surface was developed by adopting a simple electrochemical method to generate nanosized pores (∼400 nm) on the surface of SS stents. These stents when implanted in pigs showed low levels of neointima and inflammation than BMS, 3 months post-implantation.^135^ The same polymer-free nanoporous stent was loaded with the drug paclitaxel, revealing a significant reduction in neointimal hyperplasia and better endothelialization than polymer-based SES.^136^ This nanoporous stent, in another study, was spray-coated with sirolimus drug on the abluminal surface and immobilized with anti-CD34 antibodies on the blood-contacting luminal surface. This polymer-free stent completely re-endothelialized in 2 weeks with minimal restenosis *in vivo.*^137^ These nanoporous stents with anti-CD34 antibody immobilization alone facilitated effective capture of CD34+ ECs, with significantly high endothelialization.^138^ The same clinically tested platform as above, but containing CREG (a markedly upregulated gene during SMC differentiation) showed a similar degree of inhibition of SMCs as that of the drug sirolimus. However, EC proliferation was improved by CREG, in contrast to sirolimus which inhibits ECs. This CREG eluting stent attenuated neointimal formation with accelerated re-endothelialization after 4 weeks in porcine.^139^ BICARE is a novel version of the above nanoporous PFSES which elutes dual drugs (rapamycin and probucol). To assess the safety and efficacy of this PFSES-based dual drug delivery system (DDES), nanoporous SS stents loaded with probucol and rapamycin in combination were implanted in a porcine coronary artery. This DDES was found to be as safe as the commercial BMS and SES, but did not show any enhancement of re-endothelialization in porcine arteries.^140^

#### Nano-thin-films and their combination with drugs/biologics

Apart from the nanostructured topography generated on metallic surfaces, deposition of thin films on stents/substrates has also been widely examined. The concept of utilizing stent coatings was initially introduced as a means to mask the underlying stent surface, to prevent ion leaching from bare-metal stent surface into the bloodstream.^41^ Coating a stent with a thin film of biocompatible surface can improve blood compatibility, vascular cell response as well as the corrosion potential of the implant.^4,141^ These coatings can act as an inert barrier between the blood/tissue and metal with good biocompatibility.^142,143^ Deposition of thin films is a common and effective technique in surface engineering. Methods for thin-film deposition can be either physical or chemical, based on the nature of the deposition process. Chemical methods such as chemical vapor deposition (CVD), atomic layer deposition (ALD) and sol-gel involves gas- or liquid-phase chemical reactions, whereas physical methods involves sputter deposition, evaporation, and spraying.^144,145^ Such coatings can be based on polymers, inorganics, or other biocompatible materials.^146^

##### Titanium-oxide and titanium-oxy-nitride nano-thin-film coatings on stents

Titanium oxide-based coatings are the most promising coatings for cardiovascular stent applications among all inorganic materials, offering good blood compatibility, which is attributable to its surface energy and semiconducting behavior.^147,148^ The addition of nitrogen to TiO_2_ films has shown remarkable improvements in its blood compatibility due to the presence of nitride oxide on the surface.^149^ Adhesion of platelets and fibrinogen deposition were minimal for titanium–nitrideoxide (TiNOx) coatings in comparison with titanium oxide.^150,151^ TiNOx coating of thickness 500 nm was generated on metallic stents by reactive physical vapor deposition (PVD). The number of ECs on titanium oxide and titanium nitride was higher in comparison with control SS and NiTi substrates.^152^ Preclinical testing of these TiNOx-coated stents in a porcine model showed significantly less neointimal hyperplasia than SS stents at 6-weeks.^149^ The promising results from this study facilitated its transition to the clinical trial stage. Likewise, oxides of titanium, viz., Ti–O and TiO_2_, have been extensively investigated as stent coatings. A 25-nm-thick Ti–O film modified CC vascular stent developed via magnetron sputtering exhibited a faster rate of endothelialization without any thrombus, in-stent stenosis, or inflammatory reaction *in vivo.*^153^ Similarly, TiO_2_ thin-film layers consisting of embedded nanoscale TiO_2_ particles deposited on electro-polished SS using sol-gel dip coating showed enhanced blood compatibility *in vitro*, with longer blood clotting times and lesser platelet adhesion.^154^ Two types of titanium-based coatings having different thicknesses of 300 and 500 nm, respectively, deposited on 316L SS by a sol-gel route, displayed increased proliferation rates of ECs and also induced prolonged plasma recalcification time. In contrast, response to SMCs was not significantly altered by this chemistry^155^ and proved no superiority to BMS *in vivo.*^156^ In another study, a 100-nm-thick TiO_2_ film having a surface roughness of 35 nm deposited on magnesium–zinc (Mg–Zn) bioresorbable vascular scaffold helped to improve endothelial cell spreading with a good cytoskeletal arrangement. Moreover, the protective TiO_2_ layer had the potential to reduce the degradation rate of bare Mg–Zn alloy and retain its functionality.^157^ Likewise, Cu-doped TiO_2_ nanofilms containing Cu ion crystals sized 10 nm were deposited on wires through sol-gel method, wherein Cu behaved as a redox co-catalyst and promoted NO release. *In vitro* studies revealed a significant reduction of fibrinogen adsorption and platelet coverage, along with superior antithrombotic properties and anti-inflammatory ability. Reduction in neointimal thickening and suppression of inflammation, along with re-endothelialization, were noted *in vivo* within 4 weeks.^158^

**FIG. 4.**
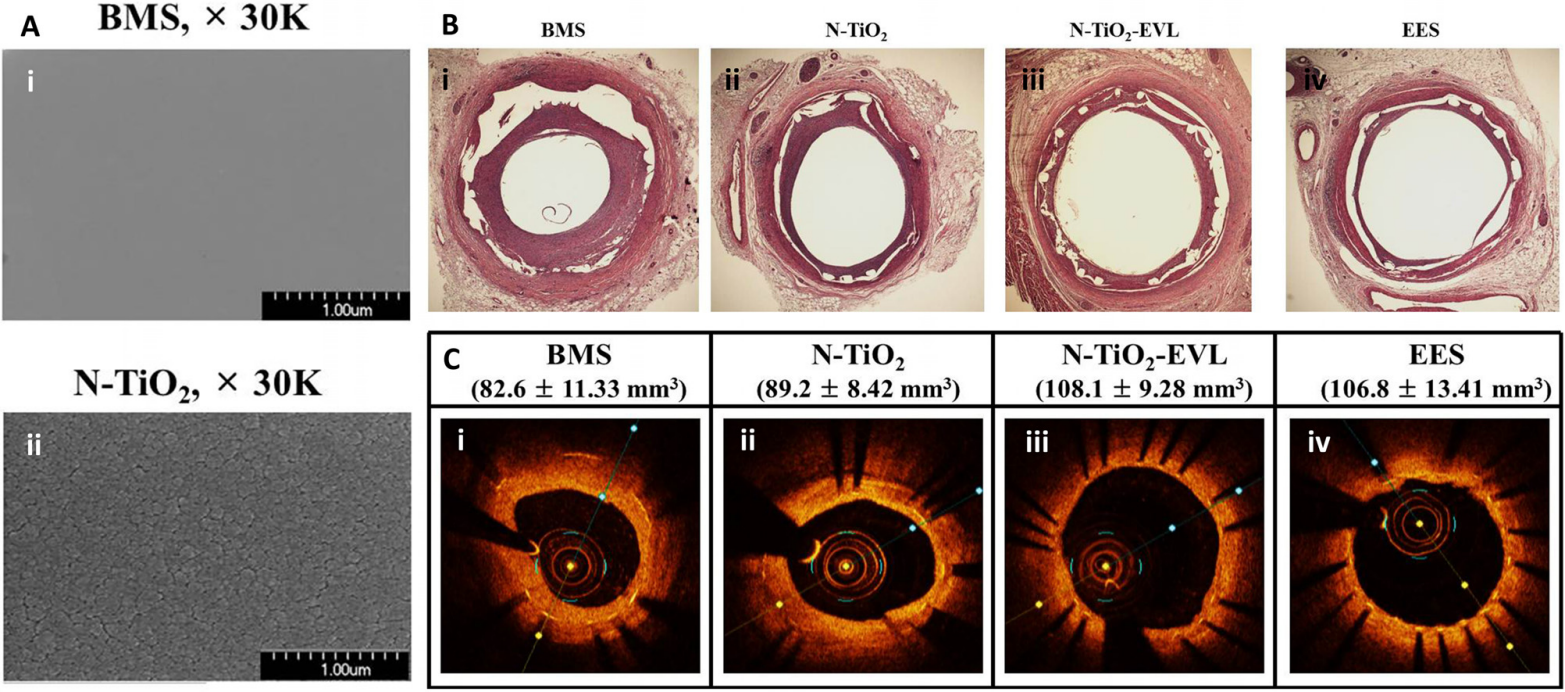
(a) SEM images of (i) BMS and (ii) N–TiO_2_ film deposited on a bare stent. (b) Histopathological H&E staining and (c) optical coherence tomography of porcine coronary arteries implanted with (i) BMS, (ii) N–TiO_2_, (iii) N–TiO_2_ with everolimus, and (iv) EES for 4 weeks. The restenosis area was significantly decreased in the N–TiO_2_-everolimus group compared to that in the BMS group and was at par with the commercial EES. Reprinted with permission from Park *et al.*, Mater. Sci. Eng.: C **91**, 615–623 (2018). Copyright 2018 Elsevier. (BMS: bare-metal stent; N–TiO_2_: nitrogen-doped TiO_2_ film; EES: everolimus-eluting stent.)

Metallic coronary stents based on CC coated with TiO_2_ by a plasma-enhanced chemical vapor deposition (PECVD) process and loaded with drugs/biologics have also been tested for their ability to improve *in vivo* response after implantation in animal models. TiO_2_ films having a surface roughness of ∼10 nm and chemically grafted with heparin were found to reduce neointima formation with decreased inflammation and fibrin deposition.^159^ A similar study by the same group also investigated the effect of polymer-free TiO_2_ film-coated stent with abciximab or alpha-lipoic acid (ALA) *in vivo*. Both the thin-film-coated stents showed an effective reduction of in-stent restenosis and accelerated re-endothelialization.^160^ A similar TiO_2_ coating on stents, but having a dual-delivery system of abciximab (drug) and Kruppel-like factor 4 (KLF4)-plasmid (gene), showed analogous results.^161^ Yet another platform developed by this group for coating drugs like tacrolimus or everolimus on stents was nitrogen-doped TiO_2_ (N–TiO_2_) thin films. Drug coating was provided on the abluminal surface by electrospraying/dip coating, while N–TiO_2_ films were deposited by PECVD. The drug coating proved to be effective in reducing inflammation and in-stent restenosis, while the titania layer helped in increased re-endothelialization and reduced thrombosis *in vivo* (Fig. 4). The study yielded results that proved the non-inferiority of their stent to commercial controls.^162,163^ A 200-nm-thick nano-TiO_2_ ceramic film was deposited by radio frequency magnetron sputtering along with EC selective adhesion peptide Arg-Glu-Asp-Val (REDV) coating (polydopamine-coating technology) on 316L SS stents, which efficiently reduced nickel ion release from SS, and also promoted EC adhesion and proliferation with increased NO release. REDV/TiO_2_-coated stents when implanted in rabbit iliac arteries effectively reduced in-stent restenosis and promoted re-endothelialization as compared to TiO_2_-coated rapamycin-eluting stent and BMS.^164^

##### Carbon-based nano–thin-film stent coatings

Diamond-like carbon (DLC) thin films have also been investigated in earlier days as a stent coating because of their outstanding mechanical characteristics, and specifically the ability to reduce platelet activation and thrombus formation. DLC films of thickness 50 nm were deposited on CC stents using the PECVD method with excellent stability and crack resistance. Plasma irradiation of these films resulted in increased functional groups on its surface, making it possible to graft polymer with drugs to develop a DES that can continuously and slowly elute drugs.^165,166^ Biocompatible Si-doped DLC films deposited on Ti6Al7Nb alloy using magnetron sputtering exhibited a positive effect on the proliferation and viability of ECs.^167^ Nanothin DLC films deposited by physical vapor deposition (PVD) on CC stents had a nanostructured surface with well acceptable hemocompatibility and anti-inflammatory properties. These stents showed effective inhibition of fibrin deposition and thrombus activation, along with an early and complete endothelial healing (30 days) and decreased neointimal proliferation at 180 days in pigs.^168^ Neointimal hyperplasia was significantly lower for DLC-coated nitinol stents after implantation in a canine iliac artery model.^169^ Another class of diamond-like nanocomposite (DLN) stent coating showed reduced thrombogenicity and minimal neointimal hyperplasia in pigs. This coating when covered with another DLC showed enhanced inflammatory reaction without any added advantage, compared to single-layer DLN coating.^170^ Another fundamental study presented strong evidence for the influence of various material and processing parameters such as surface chemistry, nanotopography, and hydrophilicity, in mediating platelet adhesion as well as cell compatibility. Three different surface chemistries provided by amorphous hydrogenated carbon (C:H), Carbon Nanotube (CNT)/DLC, and titanium boron nitride (TiB_x_N_y_) thin films of ∼100 nm thickness, deposited by magnetron sputtering technique were compared. While the hydrophilicity and nanoscale roughness of C:H and TiB_x_N_y_ films favorably influenced platelet behavior, the high roughness of CNT bundles and its hydrophobicity made the surface less thromboprotective.^171^ In another study, topography, stoichiometry, and surface properties were studied as the key parameters that regulate the thrombogenicity of a-C:H and titanium nitride (TiN_x_) nanocoatings developed by magnetron sputter deposition. By appropriate tuning of the deposition parameters (ion bombardment and content of hydrogen/nitrogen in plasma), the thrombogenic behavior of these nanocoatings could be tailored.^172^

##### Other inorganic coatings

As a biocompatible inert ceramic coating material for stents, iridium oxide has also been studied. The effect of the oxidation state of Ir_x_Ti_1−x_-oxide coatings formed on Ti substrate by thermal method showed a significant reduction in platelet adhesion and activation, rendering the surface blood compatible. Moreover, a substantial improvement in the ratio of EC/SMC count was also observed.^173^ Similarly, the antithrombogenic properties of amorphous silicon carbide (SiC) stent coating developed by chemical vapor deposition process could reduce the adhesion of platelets, leukocytes, and monocytes on the stent surface.^174^

Plasma coating using various materials has also been explored on coronary stents. Trimethylsilane (TMS) plasma coating of thickness 20–25 nm was formed on 316L SS coronary stents by direct current and radio frequency glow discharges, followed by an additional NH_3_/O_2_ plasma treatment. TMS plasma coatings imparted superior corrosion resistance to SS stents, thus hindering metallic ion release into the bloodstream.^175^ These coatings possessed good long-term chemical stability and displayed improved proliferation of ECs.^176^ SiCOH plasma nanocoatings of thickness 30–40 nm were also used to modify the surface of stents by low‐temperature plasma formed by a gas mixture of TMS and oxygen. This nanocoating showed excellent hemo‐ and cytocompatibility *in vitro.*^177^

Alumina coatings on stents have also been probed for their ability to impart antithrombogenicity and also as an inert layer that can inhibit metal ion leaching. Stents coated with sub-30 nm thick Al_2_O_3_ using plasma-enhanced atomic layer deposition (ALD) displayed improved antithrombogenicity, without altering its mechanical properties.^178^ Likewise, alumina coatings of thickness 10–20 nm deposited on NiTi by ALD technique could effectively better the corrosion resistance of NiTi, and inhibit the release of Ni atoms, thus reducing the serious problems of nickel allergic reactions.^179^

Another coating that has been investigated on clinical stents is the bioceramic hydroxyapatite, which has also been utilized as a substrate for drug loading. Porous nanothin hydroxyapatite coatings on stents were found to be stable for more than 4 months, with an *in vivo* anticipated lifetime between 9 months and 1 year for the coating, during which time the loaded drug would get released completely. A similar coating on CC stents aided the elution of low doses of sirolimus, which inhibited platelet adhesion and activation *in vitro.*^180^

##### Nanothin polyphosphazene polymer-based coating

Similar to the inorganic coatings on stents, polymeric coatings of nanothickness have also been extensively investigated. Specifically, polyzene-F (PzF) surface coating has gained immense attention because of the multifarious characteristics of this polymer, which include its biocompatibility, anti-inflammatory, and inherent thromboresistance.^181,182^ These properties can potentially help to overcome the deficits of the current clinically used DES, in patients having bleeding risk. Extensive preclinical studies including vascular cell response and platelet adhesion, followed by *in vivo* testing in various animal models were carried out on CC stents with a nanoscale PzF coating of ∼50 nm thickness. This nanocoating unfailingly exhibited minimal platelet adhesion and clotting, reduced inflammation, and accelerated endothelial healing. PzF stents have also shown significantly lower neointimal thickening, reduced thrombogenicity,^183^ and rapid healing, with complete re-endothelialization in rabbits in 1 week itself.^184^ Similar were the results when implanted in a pig coronary artery as well.^185,186^ This coating showed superior endothelial coverage than commercially available DES. This polymer coating, as a result of its excellent preclinical response, has found its place in clinical trials.

Thus, the vast literature on nanostent coatings cited above clearly underlines the impact of nanotechnology in offering the primary requisite of an ideal coronary stent, viz., its *in vivo* biological response. Table I lists the diverse nanoarchitectures and coatings developed on clinical stents and their *in vivo* outcomes.

**TABLE I.** Diverse nanostructures and thin-film coatings developed on stents which have been tested in various animal models.

Type of surface on stents	Description	Development technique	Drug/biologics	Animal model	Results	References
Nanotubular structures	Titanium dioxide nanotubes (Ti stent)	Anodization	⋯	*In vivo* rabbit iliac artery	Enhanced endothelialization and minimal in-stent restenosis	89
Nanostructures	Titanium dioxide nanoleaves (SS stent)	TiO_2_ sputter deposition followed by hydrothermal	⋯	*In vivo* rabbit iliac artery	Reduction of neointima and complete endothelialization	102
	Nanoflaky MgF_2_ film (Mg–Nd–Zn–Zr stent)	Chemical conversion treatment	⋯	*In vivo* rabbit abdominal aorta	Complete endothelial lining with minimal thrombogenicity and restenosis	110
	VSMC biomimetic patterns (SS stent)	Femtosecond laser processing	⋯	*In vivo* rabbit iliac artery	Rapid re-endothelialization in thirty days	121
	Ta implanted nanoridges (CC stent)	Target-ion-induced plasma sputtering	⋯	*In vivo* rabbit iliac artery	Minimal neointimal hyperplasia and rapid re-endothelialization	125
	Nanosized silicone filament (CC stent)		Anti-CD164 antibody	*In vivo* porcine coronary artery	Improved selective EPC capture resulting in rapid endothelial healing in 1 week	129
	Nanoporous alumina (SS stent)	Physical vapor deposition of aluminum followed by electrochemical conversion	Tacrolimus	*In vivo* rabbit iliac artery	Inhibited neointimal proliferation	131
				*In vivo* porcine coronary artery	Particle debris resulting from the cracking of ceramic coating during stent expansion resulted in increased neointimal growth and stenosis	132
	Nanoporous structures (SS stent) (Lepu Medical Technologies, China)	Electrochemical method to generate pores	Sirolimus and anti-CD34 antibody/anti-CD34 alone	*In vivo* porcine coronary artery	Endothelialization in 2 weeks with minimal restenosis	135, 136
			CREG gene		Accelerated endothelium in 4 weeks	137
			Rapamycin and probucol		As safe as BMS and SES without any significant enhancement in re-endothelialization	138
Nano-thin-film coatings	Titanium nitride coating (SS stent)	Reactive physical vapor deposition	⋯	*In vivo* porcine coronary artery	Reduced neointimal hyperplasia	147
	Ti–O film (CC stent)	Magnetron sputter deposition	⋯	*In vivo* rabbit abdominal aorta	Faster rate of endothelialization	151
	Titanium nano-thin-film coating (SS stent)	Sol-gel processing	⋯	*In vivo* porcine coronary artery	Non-inferior to BMS	154
	Copper-doped TiO_2_ nanofilms (Ti wire)	Sol-gel spin-coating	⋯	*In vivo* Rat abdominal aorta	Reduced neointimal hyperplasia and re-endothelialization in 4 weeks	156
	TiO_2_ thin films (CC stent)	Plasma-enhanced chemical vapor deposition	Heparin	*In vivo* porcine coronary artery	Reduced neointima, inflammation and fibrin deposition	157
			Abciximab/alpha lipoic acid		Effective reduction of in-stent restenosis and accelerated re-endothelialization	158
			Abciximab and Kruppel-like factor 4 gene		Reduced neointimal thickening and faster endothelialization	159
	Nitrogen-doped TiO_2_ thin films	Plasma-enhanced chemical vapor deposition	Tacrolimus	*In vivo* porcine coronary artery	Reduced in-stent restenosis and increased endothelial formation	160
			Everolimus		Decreased neointimal thickening and thrombosis with faster healing	161
	Nanothin TiO_2_ film (SS stent)	Radio frequency magnetron sputtering	REDV peptide	*In vivo* rabbit iliac artery	Reduced in-stent restenosis and promoted re-endothelialization	162
	Nanothin DLC (CC stent)	Physical vapor deposition	⋯	*In vivo* porcine coronary artery	Early and complete endothelial healing in 30 days and decreased neointimal proliferation at 180 days	166
	Nanothin DLC (NiTi stent)	Physical vapor deposition	⋯	*In vivo* canine iliac artery model	Significantly lower neointimal hyperplasia	167
	Nanothin polyzene F coating (CC stent)	Deposited from a solution and subsequently dried	⋯	*In vivo* rabbit iliac artery	Rapid healing in 1 week	182
				*In vivo* porcine coronary artery	Complete endothelial coverage and reduced neointimal hyperplasia and inflammation	183, 184

### Nanofibrous systems

Nanofibers (NF) have unique advantages as coating materials for cardiovascular stents on account of their nanoscale diameter, tunable surface morphology, flexibility, porosity, and higher length/diameter ratio. The large surface area to volume ratio of NFs permits them to be good platforms for incorporation of drugs/biologics with high drug loading.^187^ Nanofibrous matrix covered biomedical implants thus facilitate localized drug-eluting platforms, which provide sustained release of different kinds of drugs (anti-inflammatory, antithrombotic, antirestenotic) for prolonged durations at required doses^188^ and protect the vessel wall from direct metal contact.^189^ This can in turn help to reduce late stent thrombosis and restenosis risks, akin to the commercial DES. More predictable laminar flow also results from nanofiber-coated stents, thus reducing the probability of restenosis,^190,191^ but there is experimental and clinical evidence demonstrating that the covering of BMS with drug eluting polymers results in increased stent stiffness and thus modifies the mechanical properties of the stent platform,^192^ which needs to be addressed.

**FIG. 5.**
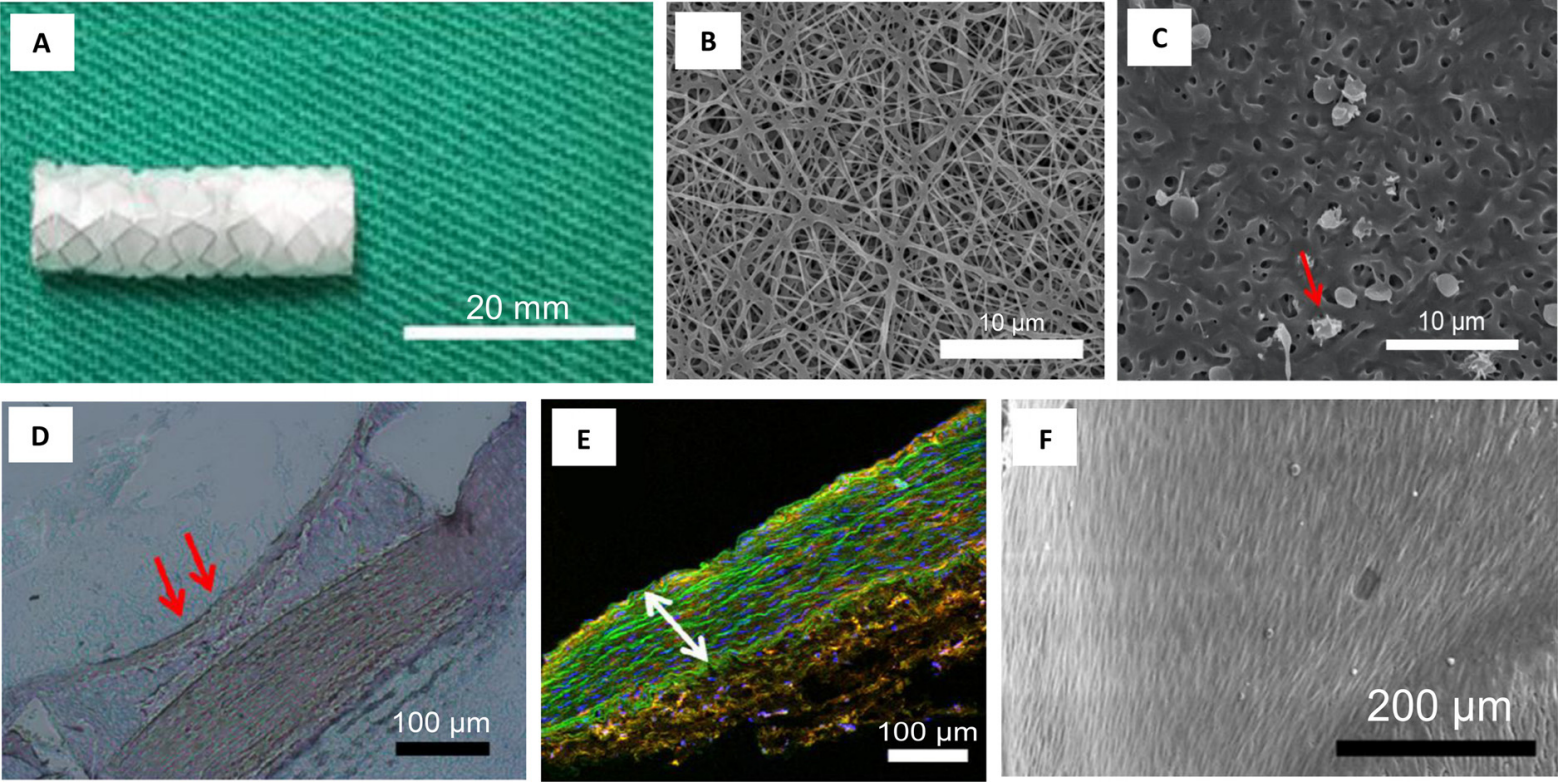
(a) Bare-metal stent with a nanofibrous membrane coating. (b) SEM micrographs of the electrospun nanofibrous membrane with ticagrelor. Magnification of ×3000. (c) SEM images of platelets on electrospun ticagrelor eluting membrane. Red arrow indicates activated platelets (scale bars = 10 *μ*m). Magnification 3000×. (d) Hematoxylin–eosin stained section of arterial lesions in ticagrelor group exhibiting a complete lining of endothelial cells (red arrows). (e) Pathological arterial lesions in the ticagrelor group stained using HES5 markers at 4 weeks following stent implantation. The amount of formed neointima suggests less proliferation of SMCs in the media. (f) SEM images of the stented vessel showing complete endothelial coverage in the ticagrelor group. Reprinted with permission from Lee *et al.*, Int. J. Nanomed. **13**, 6039–6048 (2018). Copyright 2018 Dove Medical Press.

Fibrous polymeric nanoplatforms as coatings on BMS are commonly fabricated through a simple and cost-effective technique of electrospinning.^193,194^ This is a voltage-driven fiber production process, which uses electric force to draw charged threads of polymer solutions up to fiber diameters in the order of hundreds of nanometers.^45,195,196^ Diverse biodegradable polymeric materials [e.g., poly-L-lactic acid (PLA), poly(caprolactone) (PCL), poly-lactide-co-glycolide (PLGA), chitosan (CS)], and drugs have been studied as candidates for developing nanofiber-coated stents. For example, chitosan/β-cyclodextrin nanofibers loaded with simvastatin, a drug commonly used for restenosis prevention, was developed by electrospinning to cover a self-expandable NiTi stent. Drug release time was extended by altering the duration of electrospinning and blending with cyclodextrin in the NF matrix. A dose-dependent effect on vascular cells was observed using these NF-coated stents, with EC viability affected less than SMC in the presence of the drug.^197^ The same technique was extended to polymeric stents of PLA, wherein the stents were coated with NFs of PLA/chitosan, eluting the drug paclitaxel at different concentrations. This NF-coated stent offered controlled drug release *in vitro* and displayed effective cytotoxicity to normal fibroblast cells in culture.^198^ Chitosan/PLGA-PLA nanofibers incorporating β-estradiol in Eudragit nanoparticles were developed as electrospun coatings on metallic stents, which provided high endothelial proliferation rate and enhanced NO production. Moreover, these NFs also alleviated reactive oxygen species induced toxicity to ECs *in vitro.*^190^ Another stent coating studied is the nanofibrous matrix made from a blend of PCL, human serum albumin, and paclitaxel having a coating thickness of 150–180 *μ*m and fiber diameter of ∼500 nm. After implantation in the rabbit iliac artery, these stents were less traumatic and induced weaker neointimal growth over 6 months, with increased blood flow as against that of BMS.^199^ Nanoscale cellulose acetate fibers containing rosuvastatin and heparin were developed and loaded on three different commercially available stents (SS, CC, and NiTi). This hybrid DES provided local and sustained delivery of high concentrations of the two drugs for 4 weeks, presenting a novel therapeutic method for patients who have a high risk of stent thrombosis, with minimal systemic side effects.^200^ Propylthiouracil (PTU), an antithyroid drug proven to suppress neointimal formation, was incorporated within biodegradable PLGA nanofibers with fiber diameter ranging from 112 to 622 nm and coated on BMS. These stents showed improved EC proliferation and migration, with increased NO production and eNOS activation, along with reduced platelet adhesion. A sustained release of PTU for 3 weeks, with a marked reduction in neointima formation and enhanced re-endothelialization, was observed in the injured aorta in rabbits.^201^ A blood-compatible nanofiber scaffold was developed using mesoporous silica nanoparticle (MSN) embedded PCL/gelatin electrospun nanofibers, for controlled dual delivery of 2-O-d-Glucopyranosyl-l-Ascorbic Acid (AA-2G) and heparin. Controlled release of AA-2G prevented initial oxidation and inflammation of blood cells, and the simultaneous release of heparin rendered long-term antithrombotic potential *in vitro*. Subcutaneous implantation in rats proved its biocompatibility and resistance to inflammation and thrombosis.^202^ Controlled and localized delivery of dipyridamole (DIP), a platelet aggregation inhibitor, using electrospun PCL scaffold has also been proposed as a stent coating. The released DIP accumulated in ECs without causing cytotoxicity, but inhibited EC proliferation *in vitro*, while concurrently increasing the gap junction coupling of ECs, which is a primary requirement in maintaining normal vascular physiology.^203^ In another study, PLA nanofibrous scaffolds consisting of DIP were developed by electrospinning as a cytocompatible coating for Co/Ni stents. Pharmacokinetics of the PLA/DIP nanofibers showed an initial burst, followed by a slow and controlled release for 7 months.^204^ Sustained and localized delivery of ticagrelor for about 4 weeks was achieved *in vivo* via the use of electrospun PLGA nanofiber coating on Gazelle stents. These stents reduced neointimal formation and favored endothelial recovery with lesser vasoconstrictor response and improved NO-mediated vasorelaxation after implantation in rabbit abdominal aorta as shown in Fig. 5.^205^ Likewise, PLGA nanofiber membrane-coated bare-metal SS stents were developed as a local and sustained delivery depot for acetylsalicylic acid. The electrospun PLGA/acetylsalicylic acid nanofibers had a diameter ranging from 50 nm to 8 *μ*m. This hybrid stent was highly effective as inhibitors of platelet and monocytes and promoted re-endothelialization in rabbit denuded artery. NFs induced very minimal inflammatory reaction and were completely absorbed in 4 weeks.^206^ Nanofibrous coatings of antihyperglycemic drug vildagliptin have been developed on Gazelle stents as a strategy to treat diabetic vascular disease. Stents with vildagliptin loaded PLGA nanofibers showed effective drug delivery for more than 28 days in the abdominal aorta of diabetic rabbits. This stent accelerated the revival of diabetic endothelia and also decreased neointimal hyperplasia and type I collagen content in the vascular intima than that of the non-vildagliptin-eluting group.^207^ Poly-L-lactide films cut and rolled into a cable-tie type stent were coated with rapamycin-eluting biodegradable nanofibers, which exhibited excellent mechanical properties and delivered high drug concentrations for over 4 weeks. Moreover, these stents showed a substantial reduction in intimal hyperplasia in denuded rabbit arteries during 6 months follow-up.^208^

Drug-loaded coaxial nanofibers have also been tried as stent coatings. A drug-eluting stent coated with PCL/PU blend coaxial nanofibers containing the drug paclitaxel (PTX) in PCL, with PCL as the core and PU blended PCL as the shell, has been studied for controlled drug release. PCL/PU nanofibers containing PTX inhibited the proliferation of SMCs *in vitro*.^209^ Likewise, paclitaxel/chitosan (PTX/CS) core-shell NFs have been developed on Ni-Ti stents through the co-assembly of paclitaxel and chitosan, with very high drug loading. This nanofibrous coating displayed better EC viability *in vitro* than the drug alone, due to the presence of CS outside the NFs, which prevented direct cell contact with the drug.^210^ Using a similar coaxial spinning, polyvinyl alcohol (PVA)/gelatin nanofibers (with gelatin in the shell and PVA in the core of each nanofiber) were prepared as possible stent coating. This nanofibrous scaffold possessed the requisite biocompatibility (due to gelatin), mechanical strength, and stiffness (from PVA). It promoted EC migration and proliferation, with concurrent SMC inhibition.^211^ The same group also looked into the correlation between mechanical properties and hemocompatibility of this scaffold. It was observed that the increased stiffness of coaxial nanofibers resulted in higher rates of platelet activation and thrombin formation, in comparison with individual gelatin and PVA fibers, implying that mechanical stiffness and surface roughness are dominant factors that control platelet activity.^212^

Similar to drug-loaded stents, biological molecules have also been utilized for preparing nanofibrous coatings on stent materials. In one such study, a native endothelium extracellular matrix (ECM) mimicking self-assembled peptide amphiphile (PA) nanofibrous coating functionalized with fibronectin-derived EC-specific adhesion molecule, REDV, and mussel-adhesive protein inspired Dopa residue was formed on SS surfaces. REDV–PA was designed to enhance endothelial cell-specific activity and Dopa for immobilizing REDV-conjugated nanofibers on the stent surface. *In vitro* studies proved increased adhesion, spreading, and long-term viability of ECs, with significantly lower viability of SMCs.^213^ A similar endothelial ECM mimicking nanofibrous matrix was fabricated by self-assembly of PAs containing NO donating residues, EC adhesive YIGSR peptide ligands, and enzyme-mediated degradable sites (MMP-2). A rapid release, followed by a sustained release of NO from the nanofibrous matrix for over 30 days, promoted increased EC adhesion, proliferation and concurrently limited SMC proliferation, with a 150-fold reduction in platelet attachment.^214^ Similarly, functional peptide sequences containing enzyme-mediated degradable sites combined with either endothelial cell-adhesive ligands (YIGSR) or polylysine (KKKKK) nitric oxide (NO) donors were attached to the self-assembled PAs. Linkages of two different PAs (PA–YIGSR and PA–KKKKK) to pure NO helped to develop PA–YK–NO, which was self-assembled onto electrospun PCL nanofibers to fabricate a hybrid nanomatrix. NO release could trigger significant EC activity and suppress SMC and platelet adhesion, similar to the previous study.^215^ The same group could demonstrate mitigation of inflammation due to this NO release from PA-YK-NO stent coatings under static and dynamic physiological flow conditions *in vitro.*^216^ Table II summarizes those studies that have progressed to the *in vivo* (small animal) stage.

**TABLE II.** Bare-metal stents coated with electrospun nanofibers tested *in vivo*.

Type of coating	Description	Active agent	Animal model	Results	References
Electrospun nanofibrous coatings	PCL and human serum albumin	Paclitaxel	*In vivo* rabbit iliac artery	Induced weaker neointimal growth over 6 months	197
	PLGA nanofibers on BMS	Propylthiouracil	*In vivo* rabbit injured aorta	Reduced neointimal hyperplasia and enhanced re-endothelialization	199
	PLGA nanofibers on BMS	Ticagrelor	*In vivo* rabbit abdominal aorta	Minimal neointimal formation and favored endothelial recovery	203
	PLGA nanofibers on SS stents	Acetylsalicylic acid	*In vivo* rabbit denuded artery	Promoted re-endothelialization	204
	PLGA nanofibers on BMS	Vildagliptin	*In vivo* rabbit abdominal aorta	Accelerated endothelial recovery and decreased SMC hyperplasia	205
	poly-L-lactic acid (PLLA) cable tie type stents	Rapamycin	*In vivo* rabbit denuded artery	Reduced neointimal hyperplasia	206

Despite the significant efforts undertaken on nanofiber-coated stents demonstrating controlled drug/biologics release and synchronized cell response *in vitro*, research in this direction has not progressed further to the preclinical (large animal) or clinical stages. The stability and durability of these nanofibrous coatings upon stent expansion and crimping are important aspects that need to be assessed before proposing it for clinical translation.

### Nanoparticulate systems

Nanoparticulate drug delivery systems are bestowed with significant benefits that can be readily capitalized in the cardiovascular field.^217^ This includes the possibility of target-specific drug delivery, enhanced intracellular uptake and high bioavailability, reduced drug dosage with less toxicity in tandem, tunable, and sustained drug release, etc.^218,219^ Nanoparticles (NP) loaded with drugs, when incorporated onto stent platforms, facilitate improved release kinetics and also promote a spatiotemporal delivery at the site of intervention.^220^ Nanoparticle encapsulation may also allow higher arterial wall concentrations and residence times than traditional drugs, two important factors for the prevention of restenosis.^49,219^ Additionally, drug stability is improved when loaded within a NP and an effective drug release from stents into the abluminal wall can be attained. This in turn enhances the intracellular uptake and local bioavailability of the drug at the stented site.^49,221^ After localized delivery from the stent surface, nanocarriers can infiltrate the vessel wall and form a depot, which offers a locally limited and sustained drug release into the arterial wall,^222^ thereby preventing in-stent restenosis. Local delivery of drug loaded nanoparticles, combined with antibody targeting strategies, can also permit high concentration, sustained drug therapy, which is required to prevent restenosis.^46,223^

Numerous studies have focused on enhancing localized drug delivery within arteries using nanoparticle formulations on stent platforms. Most commonly used polymers as stent coating include the bioabsorbable polymeric matrices of poly-D, L-lactic Acid (PDLLA), PLGA, PLA, PCL, etc., due to their excellent *in vivo*-biocompatibility.^224^ In one such study, sirolimus-loaded PDLLA nanoparticles exhibited good cellular and interstitial uptake as well as sufficient drug loading and revealed biphasic release kinetics with a short burst followed by a longer, slower release phase *in vitro*. However, these drug-loaded nanoparticles inhibited viability and proliferation of both EC and SMCs, although the nanodrug was less toxic to ECs compared to the free drug.^225^ A similar study in which bioresorbable PLLA stent surface was grafted with polyethylene vinyl acetate (PEVA) and PVP by plasma polymerization followed by coating with PDLLA nanoparticles carrying sirolimus displayed pronounced inhibition effect on SMCs than on ECs.^226^ Antirestenosis drugs, dexamethasone/rapamycin-loaded nanoparticles based on poly(ethylene oxide) and PLGA block copolymers, demonstrated a rapid burst release. This fast release kinetics was tuned by conjugating these nanoparticles with gelatin or albumin, which yielded a sustained release of dexamethasone and rapamycin for 17 and 50 days, respectively, after gelatin treatment.^227^ To address the problem of late stent thrombosis, an antiplatelet drug dipyridamole was loaded within PLA NPs, which showed a sustained drug release over 1 month *in vitro.*^228^ Stents coated with PLGA/chitosan nanoparticles containing a fluorescence marker (FITC) after 4 weeks of implantation in porcine coronary artery led to the specific uptake of these NPs by SMCs, yielding FITC fluorescence in the neointimal and medial layers of the stented artery in comparison with bare. However, the extent of neointima formation and re-endothelialization was comparable for the bare-metal and NP-eluting stents.^229^ This nanocarrier was utilized by the same group as a matrix for delivering various biomolecules/drugs. Imatinib mesylate [a platelet-derived growth factor (PDGF) receptor inhibitor] eluting PLGA/chitosan NPs attenuated the proliferation of SMCs associated with inhibition of the target molecule (phosphorylation of PDGF receptor-β), but showed no effect on EC proliferation *in vitro*. This observation was well reflected *in vivo* wherein a marked reduction (by 50%) of in-stent neointima formation and stenosis was observed, without any effect on re-endothelialization in pigs.^230^ Similarly, Pitavastatin loaded PLGA/chitosan NPs were as effective as the bare drug in inhibiting SMC proliferation and tissue factor expression even at very low doses. These NP eluting stents significantly reduced in-stent stenosis in a porcine model and also elicited endothelial healing effects for re-endothelialization of stented arteries.^231^ *In vivo* efficacy of a polymer-free stent that utilizes nanosized phospholipid particles to deliver sirolimus from a combined balloon-plus-stent platform was compared with a BMS and biolimus eluting stent in a porcine coronary model. Results revealed a larger lumen area with reduced neointimal thickness and stenosis, with completely covered stent struts after 28 days.^232^

Researchers have also probed into ways of selective targeting of drug/biologics to SMCs or ECs. Gene-eluting stents were developed to deliver Akt1 siRNA nanoparticles (ASNs) from a hyaluronic acid (HA)-coated stent surface to specifically suppress the pro-proliferative Akt1 protein in SMCs. This stent released Akt1 siRNA to the SMCs attached to the stent, thereby reducing cell proliferation in the implanted vasculature and also in-stent restenosis in a rabbit iliac artery model.^233^ A gene and drug co-delivering SS coronary stent coated with bi-layered PLGA NPs containing a VEGF plasmid in the outer layer and PTX in the inner core was developed. These stents could promote early endothelium healing and inhibit smooth muscle cell proliferation in the porcine coronary injury model.^234^ In another study, the possibility of using chitosan/PLGA NPs containing miR-126 dsRNA for efficient incorporation into ECs was investigated. These NPs enhanced EC proliferation and migration appreciably, while SMC proliferation was reduced *in vitro*. Implantation of stents coated with chitosan-modified PLGA NPs containing dsRNAs in a rabbit restenosis model significantly inhibited the progression of neointimal hyperplasia.^235^ Likewise, a substrate-mediated gene delivery system was prepared by using bioinspired PDA coating to which DNA complex nanoparticles, composed of protamine (PrS) and plasmid DNA encoding with hepatocyte growth factor (HGF-pDNA) gene, were immobilized. EC proliferation was specifically promoted due to HGF, with less influence on SMC growth.^236^ A nanobioactive stent platform was developed containing multiple angiogenic genes (VEGF and Ang1) carrying NPs entrapped in polyacrylic acid (PAA) functionalized CNTs and fibrin hydrogel. The developed stent coating could significantly reduce the loss of therapeutics while traversing through the vessel and during deployment, and showed significantly enhanced endothelial regeneration and inhibition of subsequent neointimal proliferation *in vivo.*^237^

**FIG. 6.**
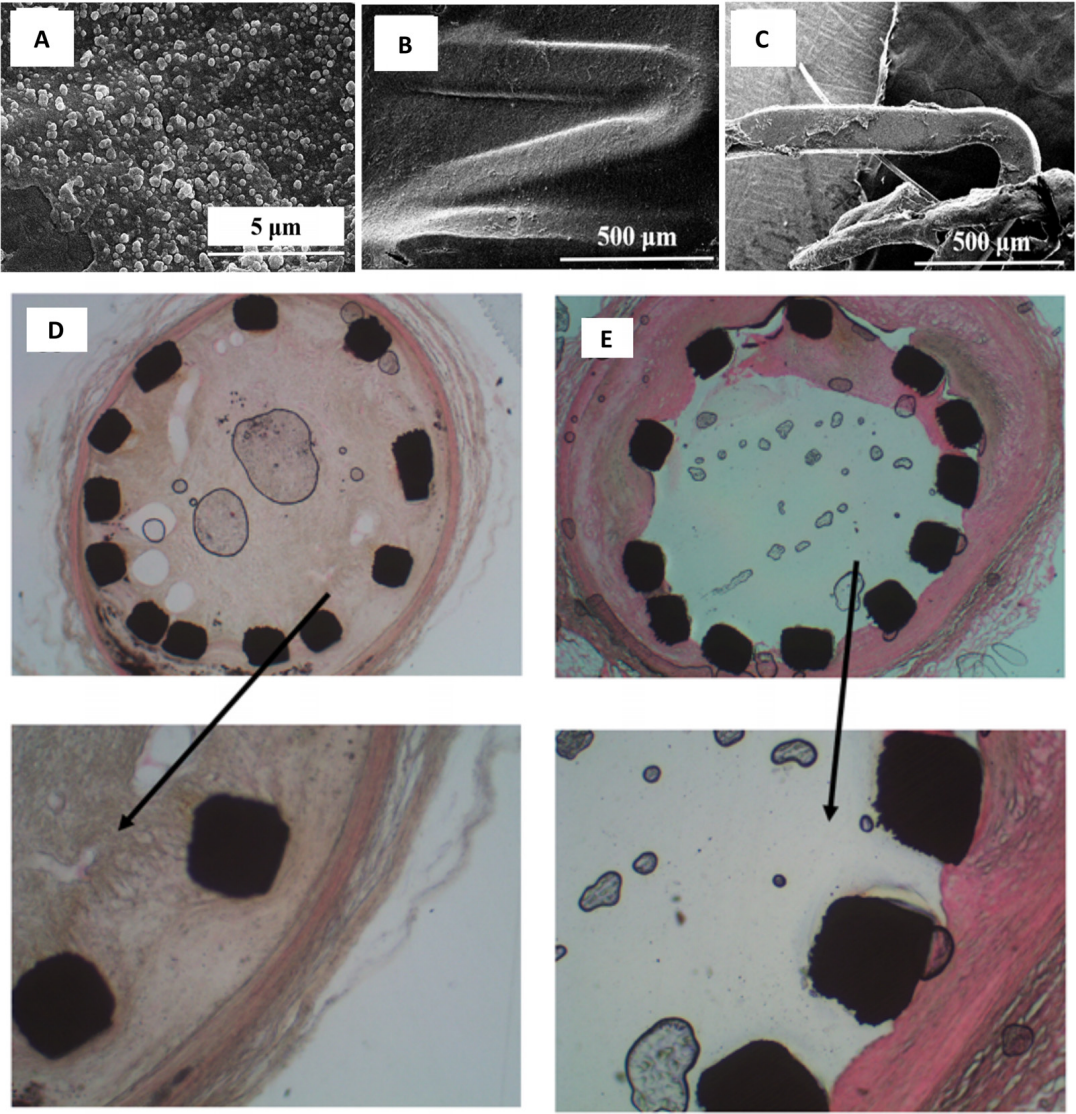
SEM images of (a) heparin/NONOate nanoparticles immobilized on polyglycidyl methacrylate (PGMA)-coated SS stents. Strut coverage on (b) SS-PGMA-Hep/NONOates and (c) control 316L SS stents harvested at 1 month. Histological hematoxylin−eosin stained images of (d) SS-PGMA-Hep/NONOates and (e) 316L SS stent after implantation for 1 month (arrows point to the higher magnification images). Reprinted with permission from Zhu *et al.*, Langmuir **36**, 2901–2910 (2020). Copyright 2020 American Chemical Society.

Heparin, an antithrombotic agent, has been utilized for developing nanocarriers that encapsulate drugs/biologics, with supplemented targeting functionality, as stent coatings. VEGF-loaded heparin/poly-l-lysine nanoparticles immobilized on dopamine-coated SS surfaces indicated enhanced blood compatibility with reduced platelet adhesion and activation, SMC inhibition, and significantly improved EC response.^238^ The same group developed a biomimetic nanocoating of laminin loaded heparin/poly-L-lysine nanoparticles, which prevented platelet adhesion and thrombus formation and showed beneficial effects in promoting EC proliferation.^239^ These nanoparticles were also loaded with fibronectin in another study to enhance the anticoagulant properties of the surface and demonstrated effective improvement in EC adhesion and proliferation.^240^ A multifunctional endothelium mimicking coating was built by cystamine-modified heparin/polyethylenimine (PEI) nanoparticles immobilized on the polydopamine surface. Active heparin along with *in situ* NO generation by the cystamine moieties in NPs resulted in good anticoagulant activity, along with a significant inhibition of SMC proliferation, promotion of EC proliferation, and tissue safety after subcutaneous implantation in rats.^241^ Similarly, a stent coating was developed by grafting Hep/NONOates onto SS stent surface. Synergistic and complementary effects of the released heparin and NO resulted in superior blood compatibility and promoted re-endothelialization with a subsequent reduction in in-stent restenosis, after implantation in the atherosclerotic rabbit model as shown in Fig. 6.^242^ Heparin coating has also been explored for its potential to minimize ion leaching from stents. Chitosan-heparin nanoparticle-coated nitinol nanotube surface helped to reduce Ni ion release and offered improved EC response. The initial burst release of heparin followed by its slow and sustained release also yielded improved blood compatibility.^243^

In addition to this, polymeric composites have also been explored for stent coatings and also as bioabsorbable stent platforms, mainly using amorphous calcium phosphate (ACP) as the ceramic component. ACP, an amorphous form of apatite with higher solubility and better bio absorbability, is capable of neutralizing the acidic by-products that are accumulated from the hydrolysis of polymers like PLGA, PLLA, etc., thereby reducing inflammation. The integration of small-dose ACP nanoparticles with PLLA resulted in the reduction of long-term chronic inflammatory response for up to 24 months after implantation in porcine arteries.^244^ SS stents coated with PLGA/ACP composites implanted into rat aortas displayed reduced restenosis with faster rates of re-endothelialization and lower inflammation. Likewise, PLLA/ACP studied as a stent platform showed significantly reduced inflammatory cell infiltration in the vessel walls of rabbit iliac arteries. No systemic toxicity was found in either PLGA/ACP or PLLA/ACP.^245^ A study on the use of PLLA/ACP scaffolds as bioresorbable stents in porcine showed larger lumen area and luminal patency rates with reduced late lumen loss and accelerated repair of the endothelium, after implantation in pigs.^246^ This fully bioabsorbable scaffold had less stent recoil and greater radial strength than PLLA scaffolds, suggesting its suitability for maintaining structural strength and functionality when implanted in porcine coronary arteries.^247^ PowerStent^®^ Absorb Bioabsorbable drug-eluting stents (BDES), fabricated by co-formulating ACP nanoparticles with PLLA and paclitaxel were developed to overcome the current limitations of BDES, viz., its biocompatibility and radial strength. ACP facilitated accelerated hydrolytic degradation of PLLA and created nanometer pores that enlarged gradually to micrometer dimensions as degradation proceeds. The increased porosity also permitted endothelial ingrowth. This BDES showed a significant reduction of in-stent restenosis, inflammation, and stent recoil in pigs.^248^ The long-term safety and efficacy evaluation of these stents in porcine revealed minimal restenosis and complete re-endothelialization, with no stent thrombosis.^249^ A fully bioresorbable PLLA/ACP nanoparticle composite scaffold containing sirolimus that could release more than 70% of drug within 28 days, and completely degrade the polymer matrix within 2–3 years *in vivo*, was also studied. This stent showed a similar safety profile as sirolimus-eluting stents, with long-term inhibition of neointimal proliferation in pigs.^250^ Another material system investigated for rendering multifunctionality to stents is nanoscale copper-based metal-organic frameworks (MOFs). Nano-Cu–MOFs of size 10–100 nm immobilized on Ti/SS wires via polydopamine coating facilitated *in situ* delivery of Cu ions, which enabled a simultaneous catalytic generation of NO. Synergistic effects of NO and Cu ions released from nano-Cu–MOF surface resulted in reduced platelet activation, SMC and macrophage suppression, and EC proliferation. Wires coated with these nano-MOFs demonstrated excellent anticoagulation, re-endothelialization, and antihyperplasia properties after implantation in rat abdominal aorta.^251^ A non-biodegradable nanocomposite polymer based on polyhedral oligomeric silsesquioxanes (POSS) nanoparticles and poly(carbonate-urea)urethane (PCU) having antithrombogenic and *in situ* endothelialization properties were studied as coatings for stents. POSS–PCU has been used in in-man trials for various other applications owing to its superior biocompatibility and unique biophysical properties.^252^ NiTi stent was deposited with POSS–PCU by electrohydrodynamic atomization to eliminate the release of toxic ions from the underlying substrate. POSS–PCU coating on NiTi stents showed enhanced peel strength, surface resistance, and biocompatibility *in vitro.*^253^ This nanocomposite polymer with covalently attached anti-CD34 antibodies was also developed as a coating for BMS to enhance the capture of circulating EPCs and promote re-endothelialization. Antibody conjugation resulted in increased EPC capture, while maintaining *in vitro* biocompatibility and hemocompatibility.^254^ The same group developed small-caliber covered stents using POSS-PCU, wherein the metal struts were fully embedded within the membrane. Platelet studies supported the non-thrombogenicity of POSS-PCU *in vitro.*^255^

A polymer-free-composite was developed with an assembly of the first layer of thin carbon nanotube (CNT) film onto SS stents, followed by a second layer of mesoporous silica nanoparticles (MMSN)/CNTs coating. This nanostructured coating exhibited excellent mechanical flexibility, blood compatibility, drug loading, and continuous drug release for up to 2 weeks *in vitro*. *In vivo* implantation of this nanostructured DES in rabbit abdominal aorta showed an early-stage endothelialization.^256^ Likewise, silver nanoparticle (AgNPs) decorated TiO_2_–NT composites formed on Ti wire promoted protein-fouling resistance, anticoagulant and anti-inflammatory property, SMC inhibition, and low toxicity to ECs. Implantation of the functionalized Ti wire in rat abdominal aortic model revealed that photo-functionalized TiO_2_, AgNPs, and Ag+ released by AgNPs synergistically suppressed inflammation, excessive SMC proliferation, and tissue hyperplasia.^257^ Table III summarizes the different types of nanoparticulate coatings developed on stents tested *in vivo* for treating in-stent restenosis.

**TABLE III.** Different types of nanoparticulate coatings developed on stents for treating in-stent restenosis, tested *in vivo*.

Type of material	Description	Active agent	Animal model	Results	References
Nanoparticle coatings	PLGA/chitosan NPs	FITC	*In vivo* porcine coronary artery	Specific uptake of the NPs by SMCs. Extent of neointima and re-endothelialization were comparable for BMS and NP-eluting stents	227
		Imatinib mesylate		Marked reduction (by 50%) of in-stent neointima formation and stenosis without any effect on re-endothelialization	228
		Pitavastatin		Significantly reduced in-stent stenosis with elicited endothelial healing effects	229
	Phospholipid NPs	Sirolimus	*In vivo* porcine coronary artery	Larger lumen area with reduced neointimal thickness and stenosis, with completely covered stent struts after 28 days	230
	Akt1 siRNA NPs	Akt1 siRNA	*In vivo* rabbit iliac artery	Reduced smooth muscle cell hyperplasia and thereby in-stent restenosis	231
	Bi-layered PLGA NPs	VEGF and paclitaxel	*In vivo* porcine injury model	Promoted early endothelium healing and inhibited smooth muscle cell proliferation	232
	Chitosan/PLGA NPs	miR-126 dsRNA	*In vivo* rabbit restenosis model	Inhibited the progression of neointimal hyperplasia	233
	NPs entrapped in polyacrylic acid functionalized CNTs and fibrin hydrogel	Vegf and Ang1	*In vivo* injured canine femoral artery	Significantly enhanced endothelial regeneration and inhibited neointimal proliferation	235
	Hep/NONOate NP	Heparin and NONOate	Atherosclerotic rabbit model	Promoted re-endothelialization with subsequent reduction in in-stent restenosis	240
Nanocomposite coatings	Amorphous calcium phosphate NPs with PLLA	⋯	*In vivo* porcine coronary artery	Reduced long-term chronic inflammatory response for up to 24 months	242
	PLGA/ACP nanocomposites	⋯	*In vivo* rat abdominal aorta	Reduced restenosis with faster rates of re-endothelialization and lower inflammation	243
	PLLA/ACP nanoscaffolds	⋯	*In vivo* porcine coronary artery	Larger lumen area and luminal patency rates with reduced late lumen loss and accelerated repair of endothelium	244, 245
	PLLA/ACP nanocomposites	Paclitaxel	*In vivo* porcine coronary artery	Significant reduction of in-stent restenosis, inflammation and stent recoil at 1month and showed minimal restenosis and complete re-endothelialization at 6 months	246, 247
		Sirolimus		Long term inhibition of neointimal proliferation	248
	Nano-Cu- metallic organic frameworks	⋯	*In vivo* rat abdominal aorta	Excellent anti-coagulation, re-endothelialization and anti-hyperplasia properties	249
	Mesoporous silica NPs/CNT	⋯	*In vivo* rat abdominal aorta	Early stage endothelialization	254
	Silver NPs decorated TiO_2_ NT	⋯	*In vivo* rat abdominal aorta	Suppressed inflammation, excessive SMC proliferation and tissue hyperplasia	255

As evinced from the literature above, innumerable reports claim the benefits of using nanobased technologies and formulations for therapeutic and/or targeted delivery of drugs/biologics in cardiovascular applications. More extensive research is warranted for translating these findings to attain clinical benefits.

## THE WAY FORWARD TO CLINICAL TRIALS

To translate a coronary stent from bench to bedside demands a multi-step process of (i) development of a viable stent prototype, (ii) functionality and toxicological evaluation, (iii) preclinical studies in large animal models, and finally (iv) clinical trials in humans.^258^ To surpass these phases and attain a clinically satisfactory product with all regulatory approvals entails a concerted effort from scientists and industry partners alike. Despite the enormous wealth of literature in this field of coronary stents utilizing nanotechnology, only a few have moved forward to the clinical phase. Majorly, the stents with inorganic coatings or polymeric thin films have emerged successful in clinical trials.

Among the inorganic coatings, the most effective products are the non-drug eluting, bioactive titania nitride oxide-coated SS stents (TiNOx), which have established significantly lower late lumen loss (LLL), angiographic restenosis, and reduced need for target lesion revascularization (TLR) than uncoated SS stents at 6 months follow-up^259^ and zotarolimus-eluting stents (ZES).^260^ Commercially available Titan^TM^ (Hexacath, France) was associated with favorable clinical outcomes. A low incidence of stent thrombosis and repeat re-vascularization at 9 months follow-up was reported from the Titan Pori registry and Multi-center Titan registry from Israel.^261,262^ Long-term follow-up revealed a better clinical outcome of TiNOX stents vs paclitaxel-eluting stents (TITAX AMI)^263^ and everolimus-eluting stents (BASE ACS),^264^ proving that it can compete with DES and share a place in the stent market. The recent TIDES-ACS trial demonstrated the superiority of this bioactive stent vs the everolimus drug-eluting stent. This stent claims advantage of requiring only a short-term dual antiplatelet treatment post-stenting.^265^ Yet another successful candidate is the Diamond-like carbon (DLC) coated stents, which exhibited excellent clinical outcomes. MOMO^®^ (Japan Stent Technology) is a drug-free CC BMS whose surface has a nanothin coating of DLC, which displayed non-inferiority over commercial BMS in a multi-center, non-randomized clinical trial. The DLC-coated stents showed low rates of target vessel failure (TVF) and angiographic restenosis.^266,267^ In contrast, the outcomes of human clinical trials on SiC-coated stents were not very satisfactory. SiC-coated stents did not exhibit any superiority over SS stents in terms of clinical and angiographic restenosis rates, despite the advantages observed *in vitro*. This is because, the direct decline in SMC growth by cytostatic drugs seems to be a crucial mechanism in the reduction of restenosis, rather than the drop in the number of platelets deposited on the stent surface.^268,269^ Likewise, inert SiO_2_ coatings on BMS after its first-in-man trial showed unsatisfactory suppression of neointimal hyperplasia.^270^

Polymeric coatings have also proven their effectiveness in clinical trials. The preclinical results of PzF nanothin coatings have translated into early convincing clinical data stating the ability of these nanocoated stents in promoting improved endothelial healing and reduced thrombosis.^271^ The COBRA Polyzene-F stent (Celenova Biosciences Inc.) satisfied all the performance goals for TVF and lumen loss at 9 months, with an excellent safety profile, rare occurrence of late myocardial infarction (low risk for MI), without any stent thrombosis.^271,272^ One-year follow-up with PzF-coated stents showed a TVF rate (Combination of all-cause mortality, myocardial infarction, or TVR) of only 12%, with no cases of stent thrombosis. This follow-up study demonstrated excellent clinical outcomes of COBRA PzF stent and compared favorably with current devices.^273^ Furthermore, various clinical trials aimed at shortening the duration of dual antiplatelet agents or administration of monoantiplatelet therapy after implantation of COBRA PzF stents to 1 month and a follow-up of 1-year have also produced excellent clinical outcomes, especially in patients with high bleeding risks.^274,275^

**FIG. 7.**
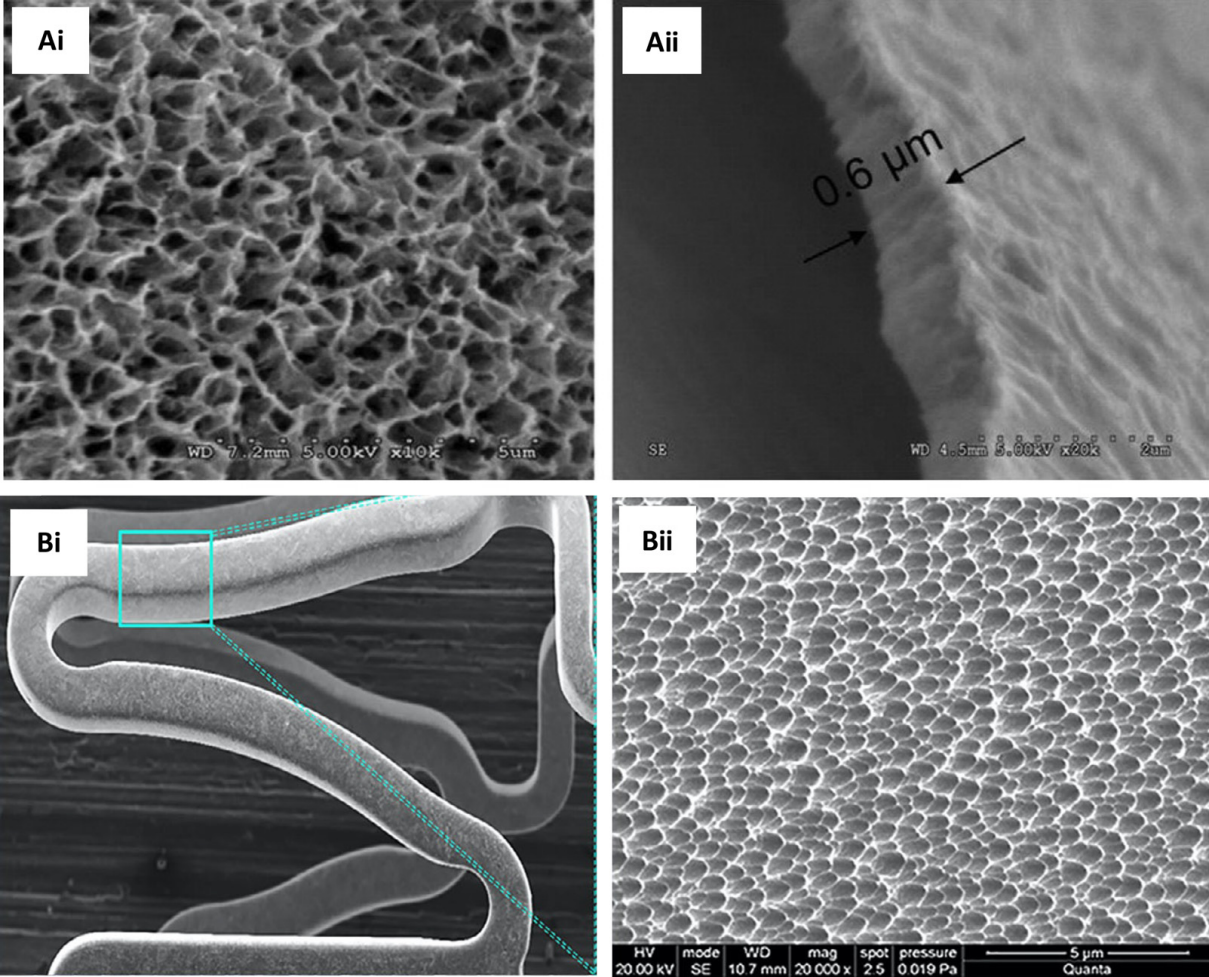
Representative SEM images of clinically tested porous stents. (a-i) Microporous hydroxyapatite-coated stent filled with sirolimus formulation (a-ii) cross section of the nanothin hydroxyapatite coating (∼600 nm). Reprinted with permission from Costa *et al.*, JACC: Cardiovasc. Interventions **2**(5), 422–427 (2008). Copyright 2008, Elsevier. (b-i) Nano+^TM^ polymer-free stent showing strut microstructure after expansion. Strut thickness is approximately 91 *μ*m having a large number of sirolimus-filled pores (∼400 nm) on the abluminal stent surface. (b-ii) Electron microscopy images of the nanopores (magnification ×20 000). Reprinted with permission from Liu *et al.*, Catheterization Cardiovasc. Interventions **95**(S1), 658–664 (2020). Copyright 2020 John Wiley and Sons.

VESTAsync™ drug-eluting SS stent (MIV Therapeutics Inc.) utilizes a polymer-free nanothin hydroxyapatite surface coating having a microporous architecture impregnated with sirolimus drug [Fig. 7(a)]. The first-in-human investigation of this stent (VESTASYNC I trial) proved it to be a feasible and safe device that elicited minimal lumen loss and neointimal hyperplasia at 4-months, with a non-significant increase up to 9 months. There were no major adverse cardiac events (MACE) within 1 year of follow-up.^276,277^ In a randomized VESTASYNC II trial in a larger group of patients, the safety and efficacy of this stent were tested with the administration of antiplatelet medication for only 3 months.^278^ A nanoporous polymer-free SS stent eluting sirolimus (PFSES) (Nano+, Lepu Medical Technology) has entered clinical trials for safety and efficacy evaluation [Fig. 7(b)]. The nanopores of size ∼400 nm are capable of controlling drug release and maintaining the mechanical integrity of the stent platform. In a 3-month follow-up study, this drug-loaded nanoporous SS stent was effective in inhibiting neointimal proliferation and promoting early vascular healing, with high strut coverage.^279^

This PFSES stent, on implantation in patients with inadequate vascular healing in 3 months, yielded complete healing at 6 months, as revealed by optical coherence tomography.^280^ In a prospective, single-blinded, multicenter, randomized clinical trial (Nanotrial) designed to investigate its safety and efficacy, nano-PFSES was non-inferior to SES at the primary end point (9 months).^281^ One-year clinical outcomes of this Nano+^TM^ polymer-free SES implantation were excellent, with low rates of target lesion failure and stent thrombosis.^282^ By utilizing the same platform, but loaded with dual drugs, viz., sirolimus and probucol, the preliminary feasibility and safety of this DDES (BICARE, Lepu Medical Technology) in the first-in-human study showed absence of any early adverse events, favorable angiographic suppression of in-stent restenosis and excellent healing at 4-months.^283^ A large-scale clinical trial of this stent in 3002 patients showed its non-inferiority to the new generation polymer-based zotarolimus-eluting stent (ZES). No differences in angiographic stenosis and late lumen loss were observed for this stent in comparison with ZES for up to 12 months, but with a lower incidence of stent thrombosis.^284^ Figure 6 displays two representative stent structures of a nanoporous, polymer-free, drug-eluting stent (Nano+) and microporous, nanothin hydroxyapatite-coated stent (VESTAsync) that are currently in the clinical trial phase.

Table IV elaborates the various stents with surface modification at the nanoscale that have entered the clinical trial phase.

**TABLE IV.** Stents with surface modification at the nanoscale in clinical trials.

Stent	Clinical trial	Clinical endpoints	Result	References
Titan (Hexacath, France) titanium nitride oxide coating	TITAX AMI	MACE (16.4% vs 25.1%) and 5-year rates of cardiac death (1.9% vs 5.7%), recurrent MI (8.4% vs 18.0%) and rate of definite ST (0.9% vs 7.1%) were significantly lower in patients with TiNOx stent compared to Paclitaxel eluting stent (PES).	Better clinical outcome of TiNOx stent vs PES in patients with acute myocardial infarction	261
	BASE ACS	At 5-year follow-up, TiNOx stent was non-inferior to everolimus eluting stent (EES) for primary endpoint of MACE (14.4% vs 17.8%). The rate of non-fatal MI was lower in TiNOx stent group (5.9% vs 9.7%) and the rates of cardiac death (2.8% vs 3.8%) and ischemia-driven TLR (8.3% vs 9.9%) were comparable for both groups.	Better clinical outcome and non-inferiority of TiNOx stent vs EES in patients with acute myocardial infarction	262
	TIDES-ACS	TiNOx stents were associated with lower rates of cardiac death (0.6% vs. 2.6%) and MI (2.2% vs. 5.0%) than everolimus eluting stent (EES) at 18 months of follow-up.	TiNOx-coated stents were non-inferior to platinum–chromium–based biodegradable polymer EES at 12 months	263
MOMO (Japan Stent Technology) Diamond-like carbon coating	Multi-center, non-randomized clinical trial	No myocardial infarction, stent thrombosis, or cardiac death were observed in patients with MOMO stent. Binary restenosis was 12.5% (n = 5), and the LLL was 0.54 ± 0.3 mm.	Safety and feasibility of MOMO cobalt–chromium carbon-coated stent	264
		No in-stent thrombosis or myocardial infarction was observed in patients with MOMO stent. The binary restenosis rate at the 6-month was 10.5 % for MOMO stent, which is lower than commercially available bare-metal stents (BMS).	Non-inferiority over commercial BMS	265
SiC-coated stent	Tenax-vs Nir-stent Study	MACE occurred in 12% of Tenax recipients and 14.3% of Nir recipients. Premature target lesion revascularization was performed in 6.9% patients in Tenax group and 5.1% patients in Nir group.	Both SiC-coated (Tenax) and non-coated (Nir) stents had low rate of MACE, with no definite superiority of any of the devices.	266
		Target lesion revascularization was performed in 2% of Tenax group and 1.6% of Nir group and subacute thrombosis was observed in 0.8% of Tenax patients.	No advantage of the SiC-coated stent over stainless steel stent with regard to clinical and angiographic restenosis rates	267
SiO_2_ coated stent	First-in-man trial	Angiographic in-stent LLL was 0.77 ± 0.44 mm, and binary restenosis occurred in 33.3% of lesions. At 12 months, cardiac death, target vessel myocardial infarction, and target lesion revascularization rate was 33.3%.	In contrast with the preclinical study, the SiO_2_ coated stent did not efficiently suppress neointimal hyperplasia in humans in this trial.	268
COBRA Pz-F stent (Celenova Biosciences Inc.) Nanothin Polyzene-F coating	One-year follow-up	Target vessel failure (composite of all-cause mortality, myocardial infarction or target vessel revascularization) rate was 12%. There were no cases of definite stent thrombosis.	The COBRA PzF stent was safe and effective and was associated with excellent clinical outcomes.	271
VESTAsync drug eluting stent (MIV Therapeutics Inc.) Nanothin-microporous hydroxyapatite surface coating	VESTASYNC I trial	In-stent LL and percentage neointimal hyperplasia were 0.3 ± 0.25 mm and 2.6% ± 2.2%, respectively, with a nonsignificant increase at 9 months (0.36 ± 0.23 mm and 4.0% ± 2.2%, respectively). There were no MACE at 1 year follow-up.	VESTAsync-eluting stent was effective in reducing LL and neointimal hyperplasia at 4 and 9 months.	274, 275
Nano+ (Lepu Medical Technology) Nanoporous polymer-free SS stent eluting sirolimus	Nanotrial	Nano+ was non-inferior to durable-polymer DES (SES) at primary endpoint of 9 months. The incidence of MACE in the Nano+ group (7.6%) was comparable to SES group (5.9%) at 2 years follow-up. The frequency of cardiac death (0.8% vs. 0.7%) and stent thrombosis (0.8% vs. 1.5%,) was low for both Nano+ and SES.	Nano+ showed similar safety and efficacy compared with SES in the treatment of patients with de novo coronary artery lesions.	279
		The 1-year Target Lesion Failure rate was 3.1% with clinically driven TLR rates (1.3%), cardiac death (1.8%) and MI (0.4%). ST occurred in 0.4% of patients.	The 1-year clinical outcomes for Nano+ polymer-free SES implantation were excellent	280
BICARE (Lepu Medical Technology) Nanoporous polymer-free SS stent eluting dual drugs sirolimus and probucol	First-in-human trial	At 4 months, angiographic in-stent late loss was 0.14 ± 0.19 mm, and the in-stent binary restenosis rate was 3.1% and complete strut coverage was 98.2%. At 18 months, TLF occurred in 9.4% patients with no adverse safety events.	The preliminary feasibility and safety of polymer-free, dual- drug eluting stent, without any adverse safety events and favorable suppression of neointimal hyperplasia.	281

## CONCLUSIONS AND FUTURE PERSPECTIVES

A way forward to advance the lacunae of the clinical drug-eluting stents is to judiciously utilize the principles of nanotechnology in the field of coronary stenting. Extensive research has been performed in this area utilizing diverse nanomaterials/surfaces that have shown effective re-endothelialization and simultaneous inhibition of in-stent restenosis. Nanothin coatings, nanotextured surfaces, and nanofibrous and nanoparticulate coatings on stents, with or without the use of active pharmaceutical ingredients, are widely explored. Specifically, those stents devoid of polymers or drugs can be a facile and cost-effective alternative to DES. *In vitro* and preclinical evaluations in small and large animal models have confirmed the utility of such nanotechnology-based stents in providing enhanced therapeutic benefits, with very few in clinical trials. The possible reasons include the risks with nanosized coatings, its flaking and thereby integrity, which needs to be confirmed before a clinical translation. Scaling up and regulatory approvals are also possible deterrents. To advance more of these stent candidates to the clinics demands surpassing the regulatory standards of functionality (especially the long-term stability and durability of nanocoatings) and toxicology as well. The promising benefits of nanomaterials science and technology would certainly help to evolve and translate these stents possessing surface modifications at the nanoscale into reality in the immediate future. In summary, nanotechnology can shape the foundation of next-generation coronary stent coatings by addressing the challenges of present-day stents.

## Data Availability

Data sharing is not applicable to this article as no new data were created or analyzed in this study.
